# Gut microbiome-associated predictors as biomarkers of response to advanced therapies in inflammatory bowel disease: a systematic review

**DOI:** 10.1080/19490976.2023.2287073

**Published:** 2023-12-03

**Authors:** Susanna Meade, Jeremy Liu Chen Kiow, Cristian Massaro, Gurpreet Kaur, Elizabeth Squirell, Brian Bressler, Genelle Lunken

**Affiliations:** a Department of Medicine, University of British Columbia, Vancouver, Canada; b IBD Centre of BC, Vancouver, Canada; c Department of Pediatrics, Univerisity of British Columbia, Vancouver, Canada; d BC Children’s Hospital Research Institute, Vancouver, Canada

**Keywords:** Inflammatory bowel disease, Crohn’s disease, ulcerative colitis, microbiome, metabolome, immunosuppressive therapy, treatment response

## Abstract

Loss of response to therapy in inflammatory bowel disease (IBD) has led to a surge in research focusing on precision medicine. Three systematic reviews have been published investigating the associations between gut microbiota and disease activity or IBD therapy. We performed a systematic review to investigate the microbiome predictors of response to advanced therapy in IBD. Unlike previous studies, our review focused on predictors of response to therapy; so the included studies assessed microbiome predictors before the proposed time of response or remission. We also provide an update of the available data on mycobiomes and viromes. We highlight key themes in the literature that may serve as future biomarkers of treatment response: the abundance of fecal SCFA-producing bacteria and opportunistic bacteria, metabolic pathways related to butyrate synthesis, and non-butyrate metabolomic predictors, including bile acids (BAs), amino acids, and lipids, as well as mycobiome predictors of response.

## Introduction

### Background

Inflammatory bowel disease (IBD) is a chronic and relapsing inflammatory condition that affects the gastrointestinal (GI) tract. Incidence is increasing and the etiopathogenesis is considered to be a combination of a genetic predisposition, environmental and dietary factors and the complex interaction between the host immune response and the gut microbiome.^1^ DNA sequencing and multiomics have dramatically advanced research into the host microbiome; alterations in which have been demonstrated in patients with IBD compared with healthy controls (HCs), termed as ‘dysbiosis’. Patients with IBD have 25% fewer taxa compared to healthy controls^2,3^ and the functional roles of the bacterial communities in IBD often favor a pro-inflammatory state.^4^

Typically, patients with IBD have reduced biodiversity with a decrease in Bacillota (previously Firmicutes [e.g. *Faecalibacterium prausnitzii* and *Roseburia hominis*]) and an increase in *Enterobacteriaceae*. Microbiota profiles differ according to IBD subtype, disease phenotype,^5^ and disease activity.^2,5–8^ As such, individualized alterations in microbial composition have been associated with future disease flare or prognostic outcomes,^9–12^ and several studies have demonstrated normalization of fecal microbiota toward that of HCs after treatment (even after adjusting for baseline degree of dysbiosis and antibiotic use).^13–15^ In the IBD mycobiome, increased Basidiomycota and reduced Ascomycota (particularly *Sacchromyces cerevisiae)* have been associated with disease activity. Differential ratios of these two phyla have been observed in disease remission, disease activity and HCs, and it has been suggested that this ratio may be a marker of fungal dysbiosis. Increased abundance of *Candida albicans* has also been observed in IBD^16,17^ with normalization after infliximab (IFX) treatment to levels observed in HCs.^16^ The mycobiome has also been correlated to the bacteriome, more so in UC than CD, and also to single nucleotide polymorphisms associated with IBD (e.g. Card9).^17^ Viruses are more prevalent in the microbiome than bacteria yet even fewer studies have investigated the IBD virome. Although the bacteriome appears to reflect IBD disease activity more accurately than the virome, several viruses have been associated with CD (e.g. *Retroviridae*). In addition, since certain viruses can modulate bacteriome activity, an understanding of the IBD virome is likely required to appreciate the relationship between microbial networks and their role in IBD pathophysiology and response to therapy.^18,19^

The loss of response to therapy in IBD has led to a surge in research on precision medicine. Several studies have investigated the association between the microbiome and disease activity, or response to therapy, in the hope that these associations may serve as biomarkers to predict therapeutic response. To date, three systematic reviews have been published investigating associations between the gut microbiota and response to therapy in IBD.^20–22^ Estevinho et al included 10 studies published between 2014–2018 largely describing the longitudinal changes in the microbiome during treatment.^20^ Radhakrishnan and colleagues reported associations between the microbiome and IBD therapy and included 19 studies, 25% of which had no baseline microbiome analysis.^22^ Jagt and colleagues systematically reviewed fecal metabolomics in pediatric IBD and only 3 of the 19 included studies compared changes dependent on response to therapy.^21^ Ananthakrishnan et al have also published a review article on microbiome biomarkers in IBD in 2020.^23^ From the available data, individual studies largely investigate the bacteriome with, or without, additional functional analysis. Since metabolomic shifts account for nearly 70% of microbiome variance, functional and metabolomic analyses provide an indirect way of quantifying microbial activity.^24^ The most commonly reported metabolic changes include a reduction in short-chain fatty acids (SCFAs), including butyrate, which coincides with the reduction in Bacillota (comprising many butyrate producing microbes),^2^ and changes in bile and amino acid profiles.^21^ There is scant data with regards to the mycobiome and virome in IBD, and even fewer studies investigating the association with disease activity or response to therapy.

### Purpose of the review

None of the described reviews specifically focused on microbiome predictors of response to advanced therapy. Sixteen of the studies included in our review,^25–40^ largely published in the last two years, were not included in the aforementioned reviews. In addition, this review focuses on the predictors of response to advanced therapy, including studies that have assessed microbiome predictors before the proposed time of response or remission. Therefore, several studies presented in previous reviews that only described associations at the time of response have been excluded. We also provide an update on the available data regarding the mycobiome and virome, which were not included in prior reviews. Therefore, this systematic review summarizes the available data on microbiome-associated predictors of response to advanced therapy for IBD.

## Methods

### Aims and primary outcome

The primary outcome was the identification of microbiome-associated predictors of response or remission to advanced therapy at any time point. We aimed to provide a qualitative summary of the literature in this area and identify the study limitations and areas for further research.

### Search strategy

We performed a systematic review of the medical literature from inception to February 2023 using Medline and Embase and searched the OVID platform. We aimed to report the microbiome predictors of response to advanced medical therapy. Search terms using subject headings and keywords included, but were not limited to, the following: ulcerative colitis, Crohn’s disease, inflammatory bowel disease, generic names for advanced IBD therapies or mechanism of action (including thiopurines, methotrexate, and licensed biologic therapies or small molecules), dietary interventions, synonyms relating to the gut microbiome and treatment outcome, response, or remission. The full details of the search string are provided in Supplementary Information 1. Hand searching of the reference lists was also performed to obtain additional studies.

### Inclusion/exclusion criteria

We included studies investigating i) patients with IBD of any age, diagnosed by conventional means, ii) with microbiome analysis (performed by any method) at baseline or before the time point for prediction, iii) related to IBD advanced therapy, iv) with clear definitions of therapeutic response, and v) comparison of responders and non-responders. We excluded abstracts, articles unavailable in English, studies investigating non-established medical therapy or ileal pouch anal anastomoses, and animal studies.

### Study selection and data extraction

Study selection was performed in two stages: title and abstract screening, and full-text review. Any discrepancies encountered during the search were resolved by the senior author (GL). Advanced therapy was defined as any of the following licensed therapies for IBD: thiopurines, methotrexate, anti-TNF therapy, anti-integrin therapy, ustekinumab, risankizumab, JAK-inhibitors, and sphingosine-1-phosphate receptor modulators. We did not include studies investigating the use of antibiotics, 5-aminosalicylic-acid (5ASA) therapy or corticosteroids in our search. 5ASA therapy is low-risk, and thus the decision to use this first line when indicated is unlikely to be altered by predictive models since if treatment is successful, immunosuppression is avoided. Antibiotics and corticosteroids are used in specific circumstances either prior to or alongside advanced therapies. Therefore, predictive markers of response have the most clinical use for advanced therapies.

Data were extracted from the selected manuscripts by the primary author (SM) using a predefined data-capture form (Supplementary information 2).

### Quality assessment

Two authors (JLCK, CM) independently assessed the methodological quality of the studies using the National Institute of Health study quality assessment tool (Supplementary information 3).^41^ The included studies were largely case series or single-arm cohort studies and therefore tools to assess for risk of bias in cohort studies with a comparator or control arm were not used. Study quality was assessed subjectively, as per the recommended guidance, based on the outcomes to 12 pre-defined questions and the overall impression after critical appraisal. If there were significant disagreement in the scoring, the authors discussed the results and reached a consensus with involvement of the primary author (SM).

## Results

### Summary of included studies

**Figure 1.**
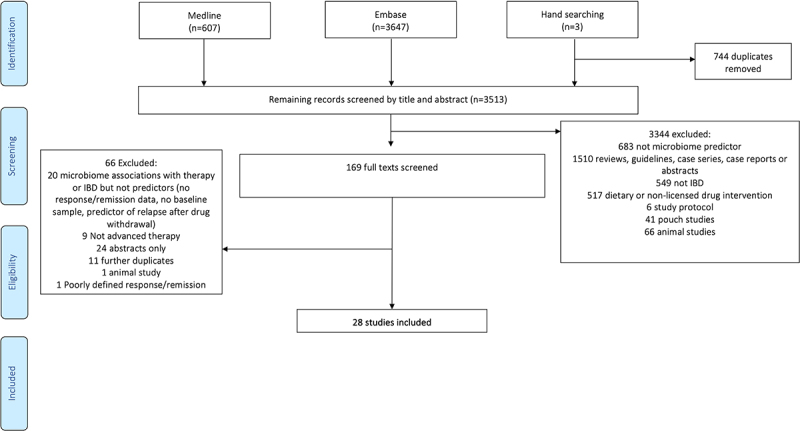
PRISMA flow diagram.

The PRISMA flow diagram displaying the study selection process is shown in Figure 1. After removal of duplicates, the Medline, Embase, and manual search of references identified 3513 articles. After screening and full-text review, 28 studies were included in the qualitative analysis.

A summary of the included studies is presented in Table 1 (with more details in Table S1). Table S2 outlines the reasons for the exclusion of certain studies that are relevant to the field. The 28 studies included one randomized controlled trial, two multicenter prospective observational cohorts, one multicenter prospective observational cohort combined with a retrospective cohort, 22 single-center prospective observational cohorts, and two retrospective observational studies.

**Table 1.** Summary of included studies.

Author Year	Study type	Primary outcome	Population	Study composition/ Microbial analysis	Responder/Non-responder with microbial analysis*	Microbial analysis method and N**	Predictive time point	Therapy	Definition of response/remission CD
Aden^14^ 2019	Prospective observational	Microbiome function/structure before and after anti-TNF	Adult	IBD: 35/170 Discovery cohort: 12 IBD (8 CD, 4 UC), 17 rheumatological, 19 HCs. Validation cohort: 23 IBD (10 CD, 13UC) on vedo/anti-TNF & 99 HCs	anti-TNF: 15R, 7NR Vedo: 11R, 2NR Discovery 9 R, 3 NR (Etanercept) Validation 6 R, 4NR (IFX) 11 R, 2NR (Vedo)	Fecal 16s amplicon sequencing, in silico metabolic modeling, inferred metabolomic function from the AGORA resource Microbial analysis: 35	Week 14	anti-TNF – IFX, Etanercept	Clinical remission CD – HBI < 5 UC – PMS < 3 Histological remission
Ananthakrishnan^42^ 2018	Prospective observational	i) Define relationship between microbial metagenomic structure and function and clinical remission with vedolizumab induction ii) identify microbiome changes on maintenance therapy iii) develop a predictive model of response to therapy	Adult	85 IBD: 42 CD, 43 UC	31R 54 NR	fecal 16s, Illumina based Shotgun metagenomic sequencing Microbial analysis: 85	Week 14	Vedolizumab	Clinical remission at week 14: CD – HBI ≤ 4 UC – SCCAI < 2. Response: Reduction in HBI/SCCAI ≥3 points
Busquets^25^ 2021	Prospective observational	Microbial signature of anti-TNF response: R vs PNR < 14W) or 2LR (cessation <12 m – LOR/AE)	Adult	38 IBD; 14 CD, 24 UC	31R, 6 NR (combined PNR and 2LR including cessation AEs)	Fecal analysis of 9 specific species by total DNA qPCR Microbial analysis: 38	0, 1, 2, 3, 6, 9, 12 months (Specific time point NR)	anti-TNF - 8 IFX, 19 ADA, 11 GOLI	Clinical remission CD – HBI ≤ 4 UC – PMS ≤ 1 and/or endoscopic remission MES ≤ 1 Biochemical remission: fCal < 250ucg/g
Chen^26^ 2022	Retrospectively observational	i) ADA efficacy and safety in Chinese patients for induction and maintenance of remission ii) Characterize fecal microbiota changes during therapy and identify potential predictors of response	Adult	115 CD	ND in subgroup with microbial analysis	Fecal 16s (Illumina amplicon sequencing) Microbial analysis: 8	12 weeks	anti-TNF – ADA	Clinical remission: CDAI < 150 Endo response: reduction in CDEIS ≥ 50% MH CDEIS 0–3
Colman^27^ 2022	Multicentre Prospective and retrospective observational (part of the REFINE cohort)	Primary: To generate a pediatric specific population PK model for vedolizumab to identify patient-specific factors affecting drug clearance as well as evaluating exposure-response relationships. Secondary: explore whether specific microbial signatures are associated with vedolizumab clearance.	Paediatric	74 IBD; 52 CD, 21 UC, 1 IBDU	40 R week 14, 41 R week 52	Fecal shotgun metagenomic analysis Microbial analysis: 13	14 weeks	Vedolizumab	Prospective: weighted PCDAI/PUCAI Retrospective; PGA CSFR fCal remission defined as < 250mcg/g
Ding^28^ 2020	Prospective observational	Metabonomic/metataxonomic predictive markers of response to anti-TNF in CD	Adult	86/99 IBD; 76 CD, 10 UC, 13 HCs	11 R, 37 NR, 28 partial R	fecal 16s metagenomic sequencing, metabolites: urine, feces and serum samples via UPLC-MS profiling analyses Samples: 57 urine, 64 serum, 48 feces 112 metabolomic patient samples	11–16 months	anti-TNF – IFX, ADA	CDAI fCal and CRP Radiological/endoscopic assessment
Ditto^34^ 2018	Prospective observation	Changes in the microbiome at baseline and 6 months after anti-TNF therapy in patients with IBD-associated spondyloarthropathy	Adult	20 IBD; 17 CD, 3 UC	ND	Fecal 16s amplicon sequencing (Illumina) Microbial analysis: 20	24 weeks	Anti-TNF -IFX or ADA	CD -HBI UC – PMS with CSFR
Doherty^43^ 2018	RCT (phase 2 CERTIFI data)	Associations between microbiota and response to ustekinumab at 6 weeks	Adult	232 CD	31 R, 201 NR	Fecal 16s amplicon sequencing (Illumina) Microbial analysis: 232	6 weeks	Ustekinumab	CDAI
Dovrolis^29^ 2020	Prospective observational	Microbial profiling and identification of predictors of response to IFX	Adult	14 CD, 6 UC, 9 HCs Received anti-TNF: 10 CD, 4 UC	7 R (5 CD, 2UC), 7NR (5 CD, 2UC)	Mucosal microbiota 16s rRNA amplicon sequencing and gene expression by RT-qPCR. 43 rectal biopsies from 29 patients. 28 pairs pre and post anti-TNF Microbial analysis: 14	12–20 weeks	Anti-TNF – IFX	CD – HBI UC – Mayo score CRP Endoscopic assessment
Effenberger^44^ 2021	Prospective observational	To assess longitudinal dynamic changes of gut microbiota in order to identify a microbial community signature of therapeutic efficacy for azathioprine or anti-TNF	Adult	65 IBD; 43 CD, 22 UC 19 CD aza, 24 CD anti-TNF, 10UC aza, 12 UC anti-TNF	32 R (12 aza, 20 anti-TNF)	Fecal 16s amplicon sequencing, FastDNA SPIN Kit Microbial analysis: 58	Weeks 12 and 30	Azathioprine, anti-TNF	CD – CDAI UC – PMPS fCal, CRP
Haberman^30^ 2019	Multicentre prospective cohort (PROTECT)	To identify gene expression and microbiota profiles that predict response to therapy in UC	Paediatric	428 UC Microbial analysis: 152	105 R (ND for subgroup with microbial analysis)	Fecal 16s RNA amplicon sequencing + genetic analysis of 206 rectal biopsies, validation in 50 patients.	4 weeks	anti-TNF, corticosteroids	Clinical scores PUCAI <10 Mild: 10–30 Moderate: 35–60 Severe: ≥65 Week 4 remission: PUCAI < 10 without treatment escalation or surgery
Hattori^31^ 2020	Prospective observational	The association between fecal microbiome and SB mucosal healing in SB CD. Secondary endpoint: association between fecal microbiome and relapse	Adult	38 CD	14 R, 24 NR	Fecal 16s rRNA amplicon sequencing (Illumina) Microbial analysis 38	44–54 weeks	Biologic and immunomodulator (not specified)	MH at baseline Endoscopic or clinical relapse (CDAI) requiring treatment change.
Hoyhtya^32^ 2022	Prospective observational	Aim to determine whether absolute abundance of gut microbes predict response to IFX	Paediatric	29 IBD: 17 CD, 6 UC, 6 IBDU	10 R, 19NR	Fecal 16s rRNA amplicon sequencing (Illumina): focus on absolute not relative abundance Microbial analysis: 29	6 weeks	anti-TNF – IFX	Validated symptom score combined with visual analogue scale + fcal < 100 = remission (95% NPV), fcal >100 NR
Kolho^45^ 2015	Prospective observational	Fecal microbiota association with disease activity and therapeutic response to TNF	Paediatric	88/94 IBD; 36 CD, 26 UC, 6 IBDU, 26 controls (8 HCs, 18 JIA)	6 R, 5NR	DNA extraction from stool and analyzed with a phylogenetic microarray (HITChip) Microbial analysis: 11	During maintenance, 3 months ahead of analysis	anti-TNF – 31 IFX, 1 ADA	PGA, PCDAI, PUCAI, fcal Responder to anti-TNF: >3fold reduction in fcal or normalization and mild or no clinical activity
Lee^33^ 2021	Prospective observational	Gut metagenome sequencing with serum metabonomics to predict response to biologic therapy	Adult	185 IBD; 108 CD, 77 UC	Clinical remission: 91 R at 14 weeks, 113 R at 52 weeks Endoscopic remission: 41 R 44NR	fecal (metagenomic sequencing, microbial profiling and functional potential) and serum (metabolomics and proteomics) Microbial analysis: 185	14 and 54 weeks	79 TNF, 21 Uste, 85 Vedo	14-week clinical remission 52 week clinical and endoscopic remission CD CR: HBI < 3 UC CR: SCCAI < 3 ER: Mayo 0–1/SESCD <3 with no ulcers
Lewis^13^ 2015	Prospective observational	Characterization of the gut microbiome in pediatric patients initiating therapy with EN or anti-TNF compared to HCs	Adult	90/116 CD; CD (52 received IFX, 38 EN), 26 HC	32 R	Fecal 16s shotgun metagenomic sequencing Microbial analysis: 86 (EN or IFX)	8 weeks	anti-TNF – 50 IFX, 2 ADA, 22 EEN, 16 PEN	Reduction of fcal to below 250mcg/g in those with a level > 250ug/g at baseline
Magnusson^46^ 2016	Prospective observational	Determine association between anti-microbial peptides and microbiota profiles in patients with UC before anti- TNF therapy and correlate these data to treatment outcome	Adult	56 UC	Faecal microbiota samples in 7 at baseline (4 R, 3NR), 15 at week 2 (8 R, 7NR), 13 at week 6 (8 R, 5NR);	Fecal qPCR Microbial analysis: 7	6 weeks	anti-TNF – 50 IFX, 6 ADA	Clinical response: decrease in total Mayo ≥3,
Mavragani^35^ 2020	Prospective observational	To explore whether interferon signature affects anti-TNF response via its interactions with microbiome	Adult	30/40 IBD; 22 CD, 8 UC, 10HC	7 R, 7NR	Amplicon 16s sequencing from colonic mucosal samples qPCR Microbial analysis: 14	12 weeks	anti-TNF – 24 IFX, 4 ADA, 2 GOLI	HBI, Mayo, CRP, colonoscopy week 0 and 12-20 wk.
Park^36^ 2022	Prospective observational	Whether microbiome changes at multiple sites can predict the effectiveness of anti-TNF in IBD	Adult	19/39 IBD; 10 CD, 9 UC, 20 HCs	ND	16s amplicon sequencing on stool and saliva and also rRNA abundance in extracellular vesicles from feces, saliva, urine, serum Microbial analysis: 19	12 weeks	anti-TNF – 11 IFX, 5 ADA, 3 GOLI	CD – CDAI UC – Mayo score or PMS
Ribaldone^47^ 2019	Prospective observational	Evaluate the microbiota at 6 months of therapy. (Secondary outcomes: association between microbiome and CRP at 6 months and predictive role on response to anti-TNF therapy.	Adult	20 CD	13 R, 7NR	Faecal 16s rRNA amplicon sequencing Microbial analysis: 20	>6 months	anti-TNF – ADA	HBI, drug persistence, CSFR
Shaw^48^ 2016	Prospective observational	Explore longitudinal changes in dysbiosis and ascertain associations between dysbiosis and markers of disease activity and response to therapy	Paediatric	19/29 IBD; 15 CD, 4 UC, 10 HCs (including 6 unaffected family members)	5 R, 12NR	Fecal 16s rRNA, amplicon sequencing (Illumina) Microbial analysis: 17	52 weeks	Immunomodulator or biologic therapy, not defined	CD – PCDAI UC – PUCAI FCal Baseline and follow up colonoscopy at about 12 months. Response determined by MH
Vatn^37^ 2020	Multicentre prospective observational	Identify fecal microbiota signatures associated with IBD and their phenotypes. Secondary outcome: identify signatures associated with disease course and treatment response	Adult	164/324 IBD; 68 CD, 84 UC, 12 IBDU, 116 Non-IBD symptomatic HCs and 44 HCs	Outcome i) Treatment escalation – 117 R, 41 NR ii) anti-TNF response at 14 weeks – 8 R, 16NR	Faecal microbial fluorescent signal strength of 54 predetermined bacterial DNA markers to provide a dysbiosis index Microbial analysis: 158 (24 with specific outcomes for anti-TNF therapy)	14 weeks	Unspecified and anti-TNF	CD – HBI UC – PMS CRP CSFR
Ventin-Holmberg^38^ 2021	Prospective observational	Predictors of response to IFX in the fecal bacterial and fungal microbiome	Adult	72 IBD; 25 CD, 47 UC	44 R (13 CD/31UC), 12 partial R (4 CD, 8 UC), 14 NR (6 CD, 8 UC)	Faecal amplicon sequencing, targeting the bacterial 16S rRNA gene and fungal ITS 1 region separately Microbial analysis 72	Week 12	anti-TNF – IFX	Endoscopy (SES-CD/Mayo score) fCal Clinical scores: HBI or PMS
Wang^15^ 2018	Prospective observational	Assess fecal microbiota changes during IFX treatment	Paediatric	11/27 IBD; 11 CD, 16 HCs	4 R, 7NR	Fecal 16s rRNA amplicon sequencing (Illumina) Microbial analysis: 11 (8 baseline samples)	After 3–6 doses	anti-TNF – IFX	PCDAI
Wang^49^ 2021	Prospective observational	Explore structure and function of micro/mycobiome and metabolome and their relationship with IFX treatment	Paediatric	29/49 IBD; 29 CD (18 received IFX), 20 HCs	11 R, 7NR	Fecal 16s rRNA/fungal ITS amplicon sequencing (Illumina) and targeted metabolomic analysis Microbial analysis: 18/24 also treated with IFX	After 3–6 doses	anti-TNF – IFX	PCDAI
Yilmaz^39^ 2019	Retrospective observational	Determine microbiota profiles according to disease phenotype, location and severity that are reproducible over the long-term	Adult	502/729 IBD: 270 CD, 232 UC, 229 non IBD	345 R, 157NR	Tissue 16s rRNA amplicon sequencing Microbial analysis: NR (reported as 4500 samples, 5 segments per patient)	5.7 years follow up	anti-TNF	QOL, disease activity, hospitalization, change in medication, adherence, surgery, CDAI, HBI, MTWAI, SCCAI, fcal,
Zhou^5^ 2018	Prospective observational (predictors) and cross-sectional study (microbiome associations with IBD vs HCs)	Identify gut microbiome patterns in Chinese IBD patients with different disease activity and status, understand microbiota profiles across different populations, clarify if any microbial biomarkers predict disease progression or response to therapy	Adult	123/196 IBD; 72 CD, 51 UC, 73 HCs (treatment response assessed in 16 CD)	9 R, 7NR	Fecal 16s rRNA amplicon sequencing (Illumina) Microbial analysis: 16	30 weeks	anti-TNF – IFX	0 and 30w endoscopy. CDAI: baseline and at infusions CRP, ESR, WCC, neutrophil ratio
Zhuang^40^ 2020	Retrospective observational	Aim to characterize fecal microbiota profiles associated with the clinical and endoscopic response to IFX at week 14 and 30.	Adult	49 CD	Clinical remission: 36 (14 weeks), 40 (30 weeks) MH: 21 (30 weeks) Endoscopic response: 39 (30 weeks)	Fecal 16s rRNA amplicon sequencing (Illumina) Microbial analysis: 49	14 and 30 weeks	anti-TNF – IFX	CDAI ileocolonoscopy at baseline and 30 weeks; MH: CDEIS 0–2

*as per study definition; **Number of patients with both microbial analysis and responder data.

IBD – inflammatory bowel disease; CD – Crohn’s disease; UC – ulcerative colitis; HBI – Harvey Bradshaw Index; MTWAI – modified Truelove and Witt activity index; SCCAI – simple Crohn’s and colitis activity index; MES – Mayo endoscopic score; PMS – Partial mayo score; R - response or remission; NR - non-response or non-remission; ND – Not documented; CDEIS – Crohn’s disease endoscopic index of severity, SES-CD – simple endoscopic score for CD; PGA – physician’s global assessment; MH – mucosal healing; rRNA – ribosomal ribonucleic acid; anti-TNF – anti-tumor necrosis factor-alpha, IFX – infliximab; ADA -adalimumab, GOLI – golimumab; aza – azathioprine; CDAI – CD disease activity index; (w)PCDAI – (weighted) pediatric CDAI; PUCAI – pediatric UC activity index; fCal – fecal calprotectin; CSFR – corticosteroid free remission; RCT – randomized controlled trial.

The studies included a total of 3447 patients; 2658 (77%) were IBD patients (52% CD), and the rest were control subjects. Of the 28 studies, sixteen analyzed IBD phenotypes (CD, UC and IBD-unclassified) together as one cohort,^5,14,25,27,32–39,42,44,45,48^ and ten^13,15,26,28,29,31,40,43,47,49^ and two^30,46^ independently reported CD and UC, respectively. There were 20 adult^5,14,25,26,28,29,31,33–40,42–44,46,47^ and 8 pediatric^13,15,27,30,32,45,48,49^ studies. Many studies performed a microbiome sub-analysis. Therefore, the data specific to microbiome predictors of response to therapy included 1232 cases who had baseline microbial analysis as well as data on therapeutic response, accounting for 46% of the study population.

Several studies investigated more than one advanced therapy, with the specific therapies investigated including anti-TNF (23 studies),^5,13–15,25,26,28–30,32–40,44–47,49^ vedolizumab (3 studies),^27,33,42^ustekinumab (2 studies),^33,43^ azathioprine (1 study)^44^ and non-specified advanced therapy (biologic/immunomodulator) in 2 studies.^31,48^

Four studies did not report specific exclusion criteria^14,42,45,46^ whilst the remaining studies excluded various patient groups (summarized in Table S1). Patients did not receive antibiotics prior to inclusion in 13 studies^5,15,25,26,29,31,36,37,40,44,47,49,50^ and in 12 studies antibiotic use was not an exclusion to enrollment.^27,28,30,32–35,38,39,43,46,48^ In addition to antibiotics, six studies also excluded probiotics (plus prebiotics in one study)^49^ if they were received between one and three months prior to enrollment.^5,29,36,40,47,49^

#### Microbiome analysis

Various techniques were used to evaluate the microbiome (Table S3). The bacteriome was evaluated in all included studies^5,13–15,25–40,42–49^ and the mycobiome in two studies (via internal transcribed spacer [ITS] amplicon sequencing).^38,51^ Fecal metagenomic sequencing was performed via 16S ribosomal ribonucleic acid (rRNA)^5,14,15,29–32,34–36,38–40,43,44,47–49,52^ or whole genome shotgun metagenomic sequencing^13,27,28,33,42^ while other studies used quantitative PCR to analyze the fecal microbiota.^25,46^ Additionally, four studies utilized rectal^29,30^ or colonic biopsies.^35,39^ Amongst studies performing microbial analysis, all assessed relative abundance with one study evaluating absolute abundance.^32^ Salivary amplicon sequencing in addition to 16S rRNA abundance in extracellular vesicles in fecal, saliva, urine and serum samples were performed in one study.^36^ Other studies assessed fecal relative abundance using fluorescent signal strength (FSS – a pre-determined primer based methodology)^37^ or HITChip phylogenetic microarray.^45^ Eight studies performed functional microbial analyses^14,26,27,29,33,40,42,44^ and four studies investigated the metabolome.^14,28,33,49^

#### Assessment of study quality

Overall the studies were of fair quality and were individually rated as good (*n* = 2), fair (*n* = 25) or poor (*n* = 1). (Supplementary information 3). Lower ratings were largely due to small sample sizes, the absence of blinding and a lack of serial outcome measurements.

#### Synthesis of results

Table S4 summarizes the microbiota and metabolomic predictors of response to therapy.

##### Microbial diversity

**Figure 2.**
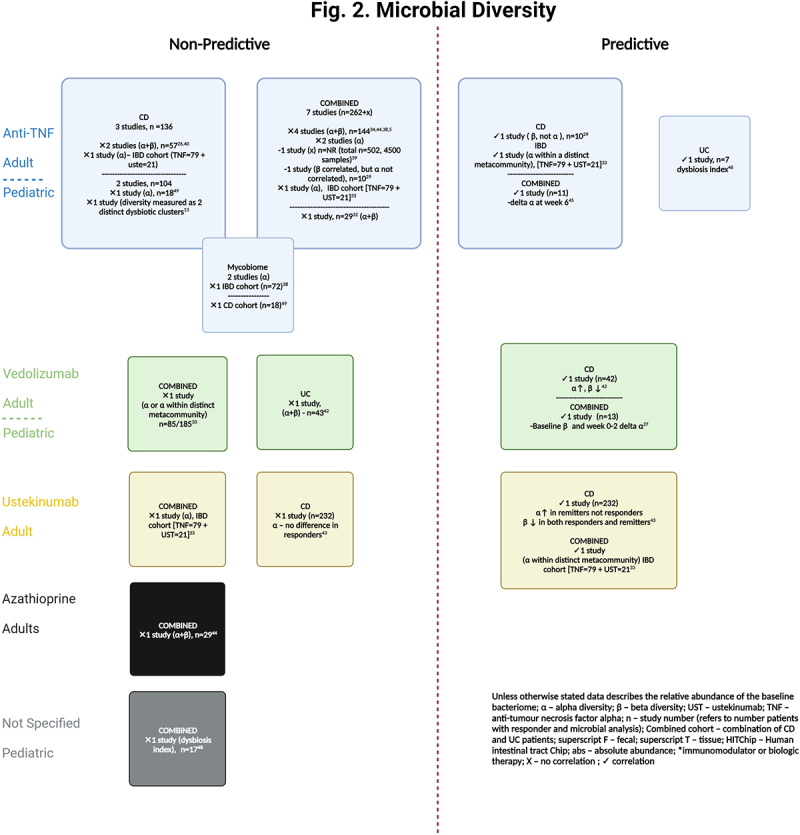
Microbial diversity.

Several studies have investigated whether alpha or beta diversity at baseline predicts response to therapy (Figure 2 and Table S5). Alpha diversity relates to variance or biodiversity within a particular sample and is often referred to as ‘evenness’ or ‘richness’. Beta diversity refers to similarities or dissimilarities in microbial communities between samples. Although fecal microbiota diversity has been shown to differ between patients with IBD and HCs^14,15,28,29,36,39,45,53^ and longitudinal changes in diversity are frequently seen after therapeutic intervention, toward eubiosis,^5,13–15,26,36,38,40,53^ this is not always the case.^37,44,54,55^

Diversity in the fecal microbiota at baseline did not predict response to therapy in 12 studies.^5,13,14,26,32,34,38–40,44,48,49^ This included one study in 19 newly diagnosed pediatric patients with IBD where remission at 1 year was endoscopically defined. The degree of dysbiosis improved over time but did not match that of HCs at final follow-up.^48^ The association between diversity and treatment response was not reported in nine studies.^15,25,28,30,31,35–37,47^ Only seven studies (mostly with small cohort sizes) observed an association, with all studies assessing the fecal microbiome. Analysis of fecal microbiota in two studies demonstrated increased alpha and reduced beta diversity at baseline. These observations predicted week-14 response to vedolizumab therapy in CD (but not UC) in 85 patients from the PRISM (Prospective Registry of IBD Study at Massachusetts General Hospital)^42^ registry (analyzed with shotgun metagenomic sequencing) and 6-week remission after ustekinumab induction in a sub-study of 232 CD patients enrolled in CERTFI (a randomized-controlled trial investigating the efficacy of ustekinumab in moderate to severe CD)[analyzed by qPCR].^43^ A further analysis of the PRISM registry demonstrating differential response to biologic therapy based on microbial richness within metacommunites at baseline is discussed in detail below.^33^ Conversely to the findings in UC patients above, Magnusson et al demonstrated that the baseline microbial diversity, evaluated with the dysbiosis index, did predict therapeutic response to anti-TNF at week-6. This study only analyzed seven baseline fecal samples.^46^ Examination of 16S rRNA from rectal tissue of 10 patients with CD prior to infliximab (IFX) induction, demonstrated differential beta diversity in responders and non-responders in both pre- and post-treatment samples.^29^ In a pediatric cohort of 68 IBD patients receiving anti-TNF therapy, Kolho et al did not directly compare responders with non-responders but demonstrated that the shift toward the eubiosis of HCs at 6 weeks (evaluated with HITChip) was predictive of biochemical remission (fecal calprotectin [fcal] <200mcg/g) 3 months later.^45^ Lastly, Colman et al demonstrated that the delta in alpha diversity between week 0–2 and the baseline beta diversity (as measured with shotgun metagenomic sequencing) predicted week-14 vedolizumab trough level. The latter was associated with corticosteroid-free remission (CSFR) at week-14. The value of this indirect association is unclear given the limited data on the benefit of vedolizumab therapeutic drug monitoring in improving long-term outcomes.^56^ Park et al published the first study investigating next-generation sequencing analysis of saliva and nanoparticle analysis of extracellular vesicles in feces, urine, saliva, and serum. No differences in diversity were observed when compared to HCs. The correlation between baseline diversity and therapeutic response was not assessed.^36^

When comparing studies based on allowance of antibiotic use at enrollment, a similar proportion of studies in each group found no association between diversity and response to therapy (6/13 and 5/12 respectively). Compared to the 18 studies that did not specifically exclude pro- or pre-biotics a similar proportion of studies found no association between diversity and response to therapy (3/6 and 8/18, respectively). Clearly, it is hard to draw conclusions from this given the heterogeneity of the individual studies, interplay of other confounding factors and lack of documentation as to whether antibiotics or probiotics were actually received in patients involved in studies where they were not an exclusion to study entry.

Given the inconsistencies in the literature and the small number of positive studies, baseline diversity does not appear to have a strong correlation with future therapeutic response. No clear trends were observed with regards to the type of diversity analysis performed and this may be at least in part due to the multiple alpha and beta diversity metrices used, reducing the ability to directly compare results between studies. Two larger studies have identified microbial networks that are associated with response to therapy.^33,39^ One of the microbial networks identified (Group 1) by Lee et al, characterized by a more diverse community profile and higher abundances of the Bacillota phyla (including *Faecalibacterium, Eubacterium, Ruminococcus* and *Roseburia*), was associated with higher rates of clinical remission.^33^ Similarily, Yilmaz et al demonstrated that a reduction in the members of cluster CD_A_ was associated with worse outcomes and poorer response to therapy.^39^ Although similar taxa were observed between cluster CD_A_ and Group 1 (*Faecalibacterium, Ruminococcus, Blautia* and *Roseburia*), microbial diversity was not observed to be higher in cluster CD_A_. Therefore, microbial interactions and the functional capacity of specific microbial networks rather than microbial diversity alone may be more important factors to focus on in future research.

#### Bacteria composition and functional capacity

Several studies have investigated the differential abundance of microbial taxa at baseline and evaluated whether they can predict response to therapy (Table S6). Where taxonomic changes do not predict response, some studies have performed functional and/or metabolomic analysis since the measurement of fecal metabolites as microbial functional properties are reported to be more consistent between individuals than the abundance of certain taxa.^14^ These additional analyses also attempt to infer causation rather than association, particularly where all identified correlations are found to be significant (i.e. taxonomic, functional, and metabolomic analyses).

#### Opportunistic organisms

In addition to the increase in Clostridia/SCFA-producing bacteria observed with therapy, several studies have observed trends in the abundance of opportunistic organisms (those with the potential to cause infection in the presence of immune dysregulation). Increased levels of *Fusobacterium* and *Escherischia-Shigella* have been noted in CD when compared to HCs in both fecal^15,29,49^ and salivary samples.^36^ Reduced relative abundance of *Enterobacter*, *Fusobacterium* and *Escherichia-Shigella* have been observed during IFX therapy compared with baseline.^40^ *Fusobacterium* have been associated with increased rates of endoscopic disease activity.^31^ Specific to anti-TNF therapy, *Fusobacterium* has been observed to fall in overall abundance after treatment,^26^ and more so in responders than non-responders.^29^ Small studies in patients with CD treated with adalimumab have observed increased abundance of *Escherichia-Shigella* in non-responders at 12 weeks^26^ and reduced abundance in responders (compared to stable abundance in non-responders) at 6 months.^47^ Effenberger et al noted an increase in abundance of *Klebsiella* in patients failing to respond to azathioprine.^44^ All of these studies observed changes at the time of the reported response with none observing baseline changes that could serve as potential predictors. Lower baseline abundance of *Escherichia-Shigella* was noted in a subset of patients achieving 6-week remission in the CERTIFI trial.^43^ An increase in baseline absolute abundance of *Actinomyces*, another potentially pathogenic organism, has also been reported in a small study of IBD patients (*n* = 29) who did not respond to IFX.^32^ In other small studies, the increased differential abundance of *Enterobacter* and *Fusobacterium* in non-responders to advanced therapy did not reach significance^48^ or conversely, an increase in *Eshcerichia-Shigella* in responders was observed after treatment (without predicting response to therapy).^29^

#### The mycobiome and virome. 

The potential of the mycobiome to predict the response to therapy has been explored in two studies. Ventin-Holmberg et al evaluated mycobiome predictors of response to IFX at 12 weeks in 68 patients with IBD. Baseline fungal diversity did not predict the therapeutic response, but an increase in *Candida* at baseline was observed in non-responders. In addition, the relationship between selected genera in the bacteriome and mycobiome correlated with response.^38^ In a smaller study including 18 patients who received IFX, disease severity correlated with certain fungal genera, but significant differences in abundance were only demonstrated after therapy.^49^ Neither of these studies replicated the findings from Sokol et al with regards to an increased Basidiomycota:Ascomycota ratio, all three of which analyzed the mycobiome via amplicon sequencing.

Only one study evaluated the virome, in addition to the bacteriome, and found no difference between IBD vs HCs.^13^ No studies were identified that have evaluated virome predictors of response to therapy.

#### SCFA-producing organisms and butyrate synthesis pathways

In a prospective observational study,^14^ 16S rRNA sequencing and fecal metabolomic profiles were examined in a discovery cohort (encompassing 12 IBD patients receiving anti-TNF therapy). Fourteen indicator phylotypes differentiated HCs from active IBD patients at baseline. *Coprococcus* and *Roseburia inulinivorans* (both SCFA producers)^57^ were the top baseline indicators, progressing from reduced abundance at baseline to no significant difference from HCs at week 30. These taxa were not associated with the response to therapy. However, in silico metabolomic analysis (the use of computational modeling to identify the predicted alterations in metabolomic pathways based on the measured abundance of bacterial species) demonstrated significant differences between IBD patients (and their therapeutic response) and HCs. In particular, a baseline reduction in metabolic interchange (a marker of metabolic exchange between organisms) was observed in anti-TNF non-remitters and remained significant after treatment. These findings were replicated in a validation cohort in which metabolomic analysis was performed on prospectively collected baseline fecal samples (23 treatment-naïve patients receiving either anti-TNF [*n* = 10] or vedolizumab therapy [*n* = 13]). Importantly, the findings persisted even after adjusting for baseline disease activity with both clinical and objective inflammatory markers. The sample size was too small to assess whether the observations were a class effect or also occurred with vedolizumab treatment. In silico meta-analysis of microbial metabolites from both the discovery and validation cohorts identified 10 baseline metabolites associated with subsequent non-remission after anti-TNF therapy when compared to HCs, seven of which were specifically reduced in non-remitters (but not remitters) at baseline (acetaldehyde, L-Arginine, Butyrate, L-lactate, ammonium, ornithine, and carbon dioxide). There was also an 81% reduction in butyrate synthesis at baseline in anti-TNF non-remitters versus remitters. To further test the hypothesis generated from the in silico modeling, they screened 50 fecal metabolites in nine patients prior to the initiation of anti-TNF therapy. The specific findings are outlined in Table S6 but highlight an increase in baseline fecal butyrate in 14-week remitters.^14^ The 12 patients with IBD (4 UC, 8 CD) in the discovery cohort were treated with etanercept and 22% remitters and 33% non-remitters were maintained on ≥20 mg prednisolone. The IBD validation cohort received licensed medications (IFX or vedolizumab), but 26% of the remitters and 50% of the non-remitters continued with varying doses of steroids at 14-weeks. Since etanercept has not been shown to be effective in CD clinical trials,^58^ the discovery cohort results may represent a response to steroids rather than to anti-TNF therapy. The validity of extrapolating data from an unlicensed drug used in the discovery cohort to a licensed drug in the validation cohort, albeit of the same therapeutic class, is uncertain. Effenberger et al also employed in silico metabolomic modelling in 65 patients with IBD and similarly identified a higher abundance of butyrate production at baseline (1.7-fold) in remitters than in non-remitters at weeks 12 and 30 (defined by CDAI < 150, normal C-reactive protein [CRP], and fcal < 150mcg/g). This was significant in the subgroup of patients with CD treated with azathioprine and not with anti-TNF therapy. No adjustment was made for baseline disease severity and therefore it is possible that increased baseline butyrate represents selection bias where certain patients with potentially milder disease activity were selected to initiate azathioprine rather than anti-TNF therapy.^44^

The IBD team at Massachusetts General Hospital has developed two prospective databases (IBD [PRSIM] and endoscopy [GI disease and endoscopy registry]) with the aim of advancing translational research in IBD with a particular focus on genetic and microbial alterations at diagnosis and also for prediction of relapse or response to therapy.^59^ Two important studies have been published based on analysis of the PRISM registry providing insights into how the microbiome may predict response to therapy. The first study^42^ evaluated data from 85 patients undergoing vedolizumab therapy and demonstrated the significance of the abundance of *Roseburia inulinivorans*, and also of Burkholderiales, which were increased at baseline in week-14 remitters (compared to non-remitters). Paired samples were available for 41 patients at both baseline and follow-up (10/24 of CD in remission and 11/17 of UC in remission). Longitudinally in CD, there was a significant reduction in *Roseburia inulinovorans* in remitters and, conversely, an increase in non-remitters. Differential abundance of several taxa were also observed after treatment, depending on the response to therapy, with associated changes in several functional pathways linked to oxidative stress, colonic inflammation, and/or host immunity (Table S6). In the few patients who had samples at all time points (baseline, 14, 30 and 54 weeks; 5 CD, 8 UC), week-14 remitters showed persistent microbiome changes at week 30 and week 54 suggesting an early microbiome ‘response’ to therapy may be associated with a durable outcome.^42^ The findings were validated in an anti-TNF cohort of 20 patients suggesting these findings were not specific to a particular therapy. The reduction in abundance of *Roseburia inulinovorans* longitudinally is at odds with the hypothesis that SCFA-producing bacteria may be beneficial. The authors postulate that this may be related to the ability of some strains of *Roseburia* to produce pro-inflammatory proteins that stimulate IL-8. This further serves to highlight the complexities in microbiome research. This same group went on to evaluate a larger cohort from the same database. 185 IBD patients underwent fecal and serum sampling prior to induction with anti-TNF, ustekinumab or vedolizumab therapy.^33^ Using microbiota profile modeling, two distinct bacterial metacommunities were identified and predicted response to therapy. Group 1 was more diverse and included *F.prausnitzii* and *Ruminococcus bromii* whereas group 2 was dominated by *Bacteroides ovatus*, *Bacteroides thetaiotaomicron* and *Veillonella parvula*. Increased microbial richness at baseline (group 1) predicted clinical remission at 1-year in patients receiving ustekinumab or anti-TNF therapy, while the same was not true for patients receiving vedolizumab. Significantly higher rates of clinical (67% vs. 36%) but not endoscopic (65% vs. 36%) remission were observed at week-52 in group 1 (compared to group 2). Group 1 patients were more likely to respond to ustekinumab/anti-TNF therapy than to vedolizumab therapy, independent of baseline disease severity or IBD subtype. Several microbial species at baseline (many of which were SCFA-producers) were associated with clinical remission at week-14 with weaker or non-significant associations noted at week-52 (Table S6). The microbial diversity of Group 1 (compared to Group 2) was negatively associated with cytokines linked to inflammatory cascades and treatment resistance, such as IL10RB and IL12RB1. When comparing anti-cytokine to anti-integrin therapy, differential serum proteins (including caspase 8 and IFNLR1) were associated with remission.^33^

The largest study to date was a retrospective analysis of 502 patients with IBD (composed of two independent cohorts). The authors identified consistent clusters of organisms associated with poor treatment response. The favorable cluster labeled CD_A_ included several SFCA-producing Clostridia (Table S6). Taxa specifically associated with response to anti-TNF therapy included *Bifidobacterium, Collinsella, Lachnospira, Lachnospiraceae, Roseburia*, and *Eggerthella*. *Phascolarctobacterium* was correlated with non-response. These findings were replicated in both cohorts. Differential abundance of taxa with regard to response to corticosteroids was also observed in both cohorts but could not be replicated in the opposite cohort. Among the 83 taxa analyzed, several were associated with disease activity (rather than prognosis): *Enterobacteriaceae* and *Klebsiella* were associated with active disease in CD, and *Prevotella* and *Ruminococcus* in UC. There was no association between CD_A_ clusters and clinical disease scores or fecal calprotectin levels. This may suggest that these parameters were poorly correlated with clinical disease activity in this study or that cluster CD_A_ could serve as a biomarker for protective physiology against disease relapse but not for disease activity.^39^

In addition to the aforementioned large landmark studies, several smaller studies have corroborated the association between SCFA-producing bacteria and response to therapy. In small pediatric studies, the presence of various genera of Clostridia in CD or combined CD/UC cohorts were associated with response to anti-TNF therapy, either clinically (pediatric CD activity index [PCDAI] ≤10 after up to 6 infusions)^15,49^ or, additionally, biochemically defined (3-fold reduction or normalization of fcal at week-6).^45^ In this latter study, severe inflammation was characterized by a reduction in butyrate-producing organisms, gram-positive bacteria (particularly *Clostridium* clusters IV and XIVa) and diversity.^45^ Wang et al also performed metabolomic analysis demonstrating that reduced levels of SCFA-producing bacteria were associated with low levels of fecal SCFAs (acetic, butyric, and propanol acids).^49^ Consistent trends have been observed in two adult studies with endoscopic assessment at the final follow-up, thus providing an association with the gold standard for objectively defined remission. A reduction of SCFA-producing bacteria predicted non-response to IFX in 72 patients with IBD (majority UC in this study) who were followed for a year (87% underwent endoscopy at final follow-up).^38^ The converse was demonstrated in 49 patients with CD, where the incremental abundance of Clostridiales from week 6 (but not baseline abundance) predicted clinical response to IFX at weeks 14 and 30.^40^ One pediatric cohort of newly diagnosed patients with IBD and endoscopically defined end points demonstrated that the differential abundance of four operational taxonomic units (OTUs) were associated with response to advanced therapy. In non-responders at baseline, *Coprococcus, Adlercreutzia* and *Dialister* were reduced and an unnamed genus of *Enterobacteriaceae* was increased. Of note, none of the UC patients responded to therapy in this study.^48^

*F.prausnitizii*, an SCFA-producing bacterium, has been investigated in several studies and deserves specific mention. Analysis of baseline fecal samples from just seven patients with UC demonstrated more pronounced dysbiosis in anti-TNF non-responders. Increased levels of *F. prausnitzii* (both at baseline and up-trending during induction) were associated with favorable outcomes. The absence of an increase in the abundance *F. prausnitzii* longitudinally during induction was associated with non-response. Additionally microbial change preceded the fall in fCal levels.^46^ Whilst *F.prausnitzii* could be a potential early biomarker for response, this finding has not been consistently replicated in other larger studies.^14,25^ The correlation has, however, been demonstrated in a large non-anti-TNF cohort. In a subgroup analysis of the CERTIFI trial investigating the efficacy of ustekinumab in the maintenance of moderate to severe CD, increased relative abundance of *Faecalibacterium* at baseline was observed in 6-week remitters and was also present at induction in every patient who entered remission at 6 weeks.^43^ In the aforementioned study from the PRISM registry, *F.prausnitizii* belonged to the metacommunity correlated with favorable response to both anti-TNF and ustekinumab.^33^ Colman and colleagues correlated the presence of *Faecalibacterium* at week-2, along with several other SCFA producing bacteria (Table S6), with week-14 vedolizumab trough levels, which were in turn associated with week-14 CSFR. In silico functional analysis in this study demonstrated the enrichment of two predominant butyrate biosynthesis pathways, which were also independently correlated with week-14 vedolizumab trough levels. They postulated that reduced levels of SCFA-producing bacteria, which are known to be associated with mucosal barrier function, may lead to increased GI drug loss. Notably, disease activity (raised erythrocyte sedimentation rate [ESR], low albumin) was also associated with drug clearance.^27^

These studies collectively investigated the connection between SCFA-producing bacteria and/or butyrate-producing pathways and their relationship with treatment response in patients with IBD. The majority of these studies indicated that reduced metabolic exchange between organisms,^14^ lower fecal butyrate concentrations^14,44^ and decreased relative abundance of SCFA- and butyrate-producing taxa^14,15,33,38,39,42,45,49^ at baseline may be predictive of non-response to advance therapies. However, inconsistency exist among the studies, particularly in the context of *F.prausnitzii*, and do not provide definitive evidence to support the use of these microbial factors as biomarkers of response. Additionally, most studies primarily focus on differences in specific taxa rather than assessing the levels of specific metabolites such as butyrate. Metabolic redundancy within the gut microbiota makes it challenging to infer that increases in SCFA- or buyrate-producing taxa directly correspond with increased levels of these metabolites without direct measurement.^60^ Future studies aiming to gain a deeper understanding of the gut microbiome’s influence on therapeutic response should extend their focus beyond taxa and consider specific metabolic pathways and the production of specific metabolites, as these microbial factors are likely to have a more direct impact on therapeutic outcomes.

#### Additional metabolomic or functional analyses

Functional and metabolomic analyses are reported to be more consistent between individuals than the differential abundance of taxa and therefore may provide a better insight into the pathogenesis of disease.^14^ In the aforementioned analysis of the PRISM registry, Lee et al performed additional functional microbiome profiling to understand the changes associated with remission. They highlighted the potential role of microbial enzymes such as glycoside hydrolases and enzymes associated with secondary bile acid (BA) synthesis. Several baseline serum secondary BAs were associated with week-14 remission and glycerophosphoethanolamines and diacylglycerols were associated with non-remission. The abundance of secondary BAs was associated with the microbiota with known 7a/B-dihydroxylation capacity (largely mediated by Clostridia). The abundance of these microbes was higher in the metacommunity which was associated with the response to ustekinumab/anti-TNF therapy. The association between response to therapy and the prevalence of microbiota with 7a/B-dehydroxylation capacity was further validated in 46 patients receiving IFX in two combined historic cohorts. Another independent cohort of 220 patients was used to corroborate the metabolomic link between raised secondary BAs, 7a/B-deyhdroxylation capacity and disease activity. Paired fecal metagenomic and metabolomic data demonstrated that patients with 7a/B-deyhdroxylation capacity had higher fecal secondary BAs. Conversely, patients with active disease had lower fecal secondary BAs, suggesting that they may be protective against inflammation.^33^

**Figure 3.**
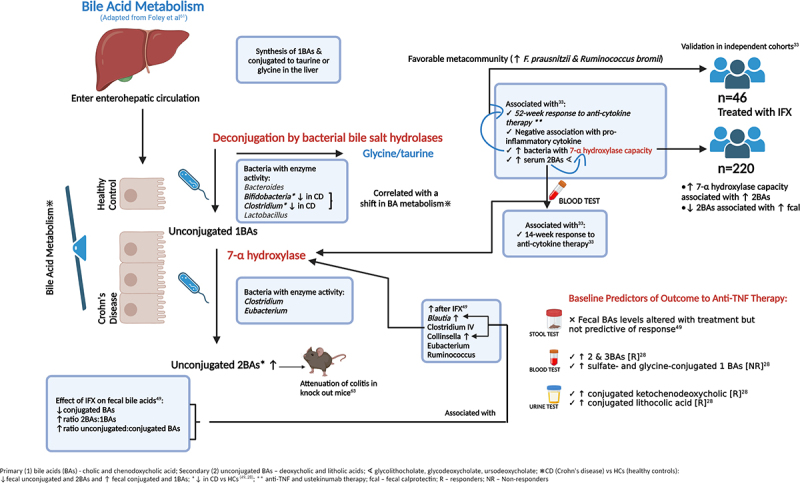
Related microbial predictors of response of therapy.

In addition to measuring fecal butyrate metabolites, Wang et al also demonstrated that CD patients at baseline compared to HCs had reduced fecal levels of unconjugated and secondary BAs and increased levels of conjugated and primary BAs (but similar overall BA levels). These findings were associated with a reduction in the abundance of *Bifidobacterium* and *Clostridium* clusters IV and XI; bacteria which are associated with bile salt hydrolase capacity, which may lead to a reduction in deconjugation. Although IFX treatment was associated with an increase in bacteria with 7a-hydroxylase activity which was associated with an increased ratio of unconjugated to conjugated BAs, fecal BAs were not predictive of response to therapy in this smaller study. The relationship between bile salt metabolism and microbiome predictors of response to therapy is summarized in Figure 3.^28,33,49,61–63^ Differential changes according to treatment response were also noted in the amino acids of the fecal metabolome (Table S6).^49^

Ding et al investigated the taxonomic and metabolomic predictors of the response to anti-TNF therapy in 86 patients with IBD. Despite no significant taxonomic changes, fecal, serum, and urinary BAs differed between responders versus non-responders, with different microbial signatures demonstrated in each sample type (Table 2 & Table S6). Increased serum secondary and tertiary BAs were similarly observed in anti-TNF responders, whereas higher levels of sulfate- and glycine-conjugated primary BAs were noted in non-responders. Including the three most predictive BAs in a composite model provided an AUC of 0.81 (±0.17) for predicting anti-TNF response. Fecal and serum histidine, urinary cysteine, and differential levels of lipid markers also predicted response. In responders, serum phosphatidylcholine, ceramide, sphingomyelin, and triglyceride levels were reduced, whereas fecal phosphocholines and triglycerides were increased. Fecal lipid profiling provided an AUC of 0.94 (±0.10) for response to therapy.^28^

**Table 2.** Predictive models.

Author	Predictive time point	Therapy	Predictive Model
**Microbial predictors**			
Busquets^25^	Samples at 0, 1, 2, 3, 6, 9, 12 months (Specific time point NR)	anti-TNF - 8 IFX, 19 ADA, 11 GOLI	They did not demonstrate response prediction with specific species as predictors of response (9 evaluated) but the ratios of four of these combined together in a model did predict response. Relative abundance of: *F. prausnitzii/Eubacteria;* *F. prausnitzii phylogroup 1/Eubacteria;* *Methanobrevibacter smithii/Eubacteria;* *Ruminococcus/Eubacteria;* Sensitivity 93.3%, specificity 100%, PPV 100%, NPV 75% Prediction of anti-TNF response. Unclear at what time point serves as predictor
Kolho^45^	During maintenance, 3 months ahead of analysis	anti-TNF – 31 IFX, 1 ADA	*Bidifidobacterium* and *Clostridium colinum* for prediction of response to anti-TNF at 6 weeks: Sn, Sp, PPV, NPV all 1 *Eubacterium*: Sn, Sp, PPV, NPV all 0.80 *Clostridiales*: Sn 0.83, Sp 1.0, PPV 1.0, NPV 0.8 *Strep. mitis* and *Vibrio*: Sn 1.0, Sp 0.83, PPV 0.80, NPV 1.0 Not direct comparison of responders with non-responders but the shift toward the eubiosis of HCs at 6 weeks was predictive of biochemical remission (fCal <200ucg/g) 3 months later Increased *Clostiridum sphenoides* and *Haemophilus spp* associated with fCal < 200ucg/g 3 months later (AUC 0.88)
Shaw^48^	52 weeks	Immunomodulator or biologic therapy, not defined	Relative abundance of 15 weighted genera, (largely of the Clostridiales family): AUC 76.5%, prediction error in responders (20%) and non-responders (25%). All 4 UC patients were non-responders
Ventin-Holmberg^38^	Week 12	anti-TNF – IFX	Selective genera used in a predictive model for 12-week remission: AUC 0.80 for all IBD patients, 0.84 for CD, 0.79 for UC Increased accuracy when genera with differential abundance in IBD subtypes included in the model: AUC 0.93 for CD (Bifidobacterium, Rothia, Atopobium, Gemella, Pseudoflavonifractor, Sutterella and Pseudomonas) AUC for UC 0.82 (Enterococcus, Clostridium, Peptostreptococcus, Faecalibacterium and Candida.)
**Combined microbial and clinical models**			
Ananthakrishnan^42^	Week 14	Vedolizumab	‘VedoNet’ Modelled in vedolizumab (*n* = 21) and validated in anti-TNF cohort (*n* = 20) AUC 0.87 (>80% true discovery rate, <25% false discovery rate) Variables: Clinical: IBD type, sex, smoking status, age at diagnosis, disease activity at baseline (HBI/SCCAI), disease duration, CRP, ESR, WCC, HB, HCT, PLT, ALB, microbiome composition Bacterial: *Roseburia_inulinivorans, Bifidobacterium_longum, Ruminococcus_gnavus, Veillonella_parvula, Lactobacillus_salivarius, Eggerthella, Burkholderiales_noname* and 33 microbiome functional pathways Prediction of 14-week remission to vedolizumab. Validated in a prospective cohort of 20 patients treated with ant-TNF (14 CD, 6UC), 13 of whom achieved remission at week-14. VedoNET correctly predicted 11/13 cases
Lee^33^	14 and 54 weeks	79 TNF, 21 Ustekinumab, 85 Vedolizumab	21/185 patients had available clinical, metagenomic, metabolomic and proteomic data AUC for predicting 14-week response after anti-cytokine therapy 96.3% (95% CI 0.88–1.00).
Doherty^43^	6 weeks	Ustekinumab	Predicting clinical remission at 6 weeks: Clinical data: age, sex, baseline steroids, BMI, disease duration and location, fcal, fecal lactoferrin, CRP, bowel stricture, CDAI subscores Microbial data: OTU relative abundance (*Dialister, Clostridium XI, Coprobacillus, Fuminococcaceae, Ruminococcus, Clostridiales, Coproccoccus, Faecalibacterium, Pasteurellaceae Escherichis/Shigalla*), alpha diversity AUC Clinical data: 0.63 (Sp 0.80, Sn 0.45) Microbial data: AUC 0.84 (Sp 0.77, Sn 0.81) Combined: AUC (0.84 (Sp 0.83, Sn 0.77) Predicting clinical response at 6 weeks: Clinical data: AUC 0.61 (Sp 0.54, Sn 0.72) Microbial data: AUC 0.76 (Sp 0.56, Sn 0.88) Combined: AUC 0.73 (Sp 0.72, Sn 0.68) Post-hoc analysis of 232 patients from CERTIFI, similar in accuracy to using combined predictive models of microbial and clinical data. This was true for both responders and remitters at 6-weeks
Zhou^5^	30 weeks	anti-TNF – IFX	*Clostridiales (Lachnospiraceae), Anaerostipes, Bacteroidales (Bacteriodaceae), Bacteroides,Clostridiales (Veillonellaceae), Veillonella (Dispare), Lactobacillales (Streptococcaceae), Streptococcus (Anginosus)* Microbial data alone: AUC 87% Combined with FCal, CDAI: AUC 0.94 CDAI and fcal alone offered accuracy of 59% and 63%
Zhuang^40^	14 and 30 weeks	anti-TNF – IFX	Incremental abundance of *Blautia* and *Lachnospiraceae* from week 6 predicts: Clinical remission at week 14 (AUC 83% [71–96%]) and week 30 (AUC 84% [72–97%]) Endoscopic efficacy at week 30 (accuracy 89% [79–99%]). Microbial markers also predicted response better than using clinical data alone. Combined with albumin and CRP predicts endoscopic response at week 30 (AUC 91% [82–99%]).
**Metabolomic modeling**			
Ding^28^	11–16 months	anti-TNF – IFX, ADA	Predictive metabolomic profiling for CD at 11–16 months after anti-TNF. Three fecal BAs with strongest association predicted anti-TNF response with AUC of 0.81 (±0.17). Five serum BAs (AUC 0.74 ± 0.15) Urine BAs (AUC 0.70 ± 0.17) Combination of above did not improve prediction Urinary cysteine predicting response: AUC 0.78 ± 0.12. Fecal/serum histidine poorly predictive Serum lipids: AUC 0.78 (±0.12); Sn 0.92, Sp 0.61 Faecal lipids: AUC 0.94 (±0.10); Sn 0.81, Sp 0.64
**Microbial profiles associated with mucosal healing predictive of response**			
Hattori^31^	44–54 weeks	Biologic and immunomodulator (not specified)	A microbial predictive score for mucosal healing was developed as a point system using these six genera. The AUC was 0.80 (sensitivity 0.64, specificity 0.92) Multivariate regression analysis adjusting for age, sex, smoking history, BMI, CDAI, CRP, ESR albumin, immunomodulator, biologic therapy, elemental diet and PPI demonstrated the microbial composite score to be the strongest predictor for MH (OR 37.5 [3.41–411.99], *p* = 0.003) Presence of these genera associated with reduced cumulative rate of relapse at 1 year Patients with a higher abundance of these bacteria were also significantly less likely to relapse over 11–13 months follow up with no relapses at final follow up in patients scoring ≥5 (0 vs 40% relapse rate for MPS ≥5 and < 5 respectively). Combining MPS < 5 with serum albumin (threshold <41.5 g/L) differentiated patients further into moderate and high risk of relapse at final follow up (20% vs 40% respectively).

HBI – Harvey Bradshaw Index; SCCAI – Simple Crohn’s and Colitis activity index; CDAI – CD activity index; CRP – C-reactive protein; ESR – Erythrocyte sedimentation rate; WCC – White cell count; Hb – Haemoglobin; HCT – hematocrit; PLT – platelet; ALB – albumin; anti-TNF – anti-tumor necrosis factor-alpha; IFX – infliximab; GOLI – golimumab; ADA – adalimumab; NR – not reported; CD – Crohn’s disease, UC – ulcerative colitis; BA – bile acids; PPV – positive predictive value; NPV – negative predictive value; Sn – sensitivity; Sp – specificity; fCal – fecal calprotectin; AUC – area under the curve.

### Studies showing no association between the gut microbiome and response to therapy

Several studies have not observed that microbial abundance is capable of predicting the response to therapy. These findings may be true, or due to several factors: the small sample size,^26,47^ how the response was defined, cohort heterogeneity or the methodology. Busquets et al included cessation due to adverse event in their definition of response and found no individual taxa associated with response to therapy.^25^ Vatn et al performed a multicentre study in Europe recruiting newly diagnosed patients with IBD to identify fecal microbiota signatures associated with IBD and their phenotypes. The secondary outcome was the identification of signatures associated with disease course and response to therapy. A total of 158 patients had information on their medical trajectory, with patients being followed for at least 12 months, 41 of whom required treatment escalation for disease flare (biologic, cyclosporine, or surgery). Disease severity at baseline according to CRP or fcal was associated with the need for escalation, but no microbial predictors were identified. This was the only study to evaluate bacterial abundance based on the FSS of 54 pre-determined bacterial DNA markers. FSS was available in 24/29 patients who escalated to anti-TNF therapy, eight of whom achieved clinical and biochemical remission. No differences were seen in this subgroup.^37^ Several of the negative studies, in terms of taxa predicting response to therapy, combined IBD subtypes^14,28,37,44^ or had a more stringent definition of clinical response to therapy requiring either at least one objective marker of inflammation or corticosteroid free remission.^26,28,44,47^ In one study, all of the non-responders (7/20) were in clinical remission but failed to meet the criteria for response due to ongoing corticosteroid therapy.^47^ While this study is likely underpowered, the inflammatory burden in each group was not compared objectively and differential abundance of bacteria has been observed with corticosteroid exposure.^32,43^ In addition, samples were taken at baseline and 6 months.^47^ Larger studies performing sequencing more frequently have demonstrated a predictive value at 14- but not 52-weeks with regard to specific taxa.^33^

### Predictive models

The correlation between microbial biomarkers at baseline and subsequent outcomes after treatment are just associations. As previously described, true predictors need to be compared between remitters and non-remitters and performance should be evaluated as predictors (sensitivity, specificity, positive and negative predictive values) rather than just a significant p-value for association.^64^

In addition to the metabolomic model from Ding et al,^28^ ten other studies have developed predictive scores for potential microbiome predictors of the response to therapy. These results are outlined in detail in Table 2. Four studies demonstrated that microbiome predictors alone could predict the response to therapy, with an AUC of 77% or higher. In three small studies, the relative abundance of varying numbers of genera (often Clostridia) predicted the clinical response to anti-TNF during, or shortly after induction,^25,38,45,48^ (although in one of these studies, the time point of the predictor is unclear).^25^ One study included specific fungal genera with differences in abundance between IBD subtypes, thus including *Candida* in the UC model. This improved the accuracy for predicting 12-week response to IFX to 93% for CD and 82% for UC.^38^

Five studies combined clinical parameters (three of which incorporated fecal calprotectin^5,43,45^) with microbial variables and demonstrated that the addition or sole use of microbial variables predicts response to therapy better than using clinical data alone. Doherty et al were able to predict 6-week response to ustekinumab in the CERTIFI sub-study.^43^ In the PRISM registry a combined microbial model (‘vedoNet’) that included 40 variables (taxa and metabolomic pathways) predicted 14-week clinical response to vedolizumab (AUC 87% and a false discovery rate of less than 25%). This was validated in a prospective cohort of 20 patients treated with anti-TNF and correctly predicted response in 11/13 cases.^42^ In the extended analysis of the PRISM registry data 21 patients with available clinical, metagenomic, metabolomic and proteomic results were evaluated. Their proposed model predicted response to anti-cytokine therapy with AUC 0.96 (95% CI, 0.88–1.00). Limiting factors include the use of clinical remission rather than objective markers in over 50% patients, the combination of ustekinumab/anti-TNF as ‘cytokine responders’ and only 21/185 patients being included in the formation of their predictive model.^33^ In 49 patients with CD treated with IFX, the trend in microbial composition (particularly *Blautia* and *Lachnospiraceae*) from baseline to 6 weeks predicted clinical remission at weeks 14 and 30 (accuracy 83% [71–96%] and 84% [72–97%], respectively) and endoscopic response at week 30 (accuracy 89% [79–99%]). No differences were observed between responders and non-responders at baseline.^40^ In a subset of 16 patients with baseline and follow-up microbial profiling, restoration of Clostridiales toward that of HCs was associated with clinical remission, and patients with a higher abundance at baseline responded better to therapy (accuracy of 87%, improving to 94% if combined with clinical parameters).^5^

Lastly, Hattori et al investigated the association of the microbiome with mucosal healing (MH) and whether the microbiome could predict prognosis. They performed baseline fecal 16S rRNA sequencing in patients undergoing entire small bowel (SB) endoscopic assessment (either by video capsule endoscopy or double balloon enteroscopy) in 38 patients with SB CD receiving immunomodulators or biologic therapy. Patients with active colonic inflammation or perianal disease were excluded from this study. A low relative abundance of Bacteroidetes and higher *Fusobacterium* was observed in patients with active endoscopic SB disease. *Faecalibacterium*, *Lachnospira, Paraprevotella, Dialister, Streptococcus* and *Clostridium* were reduced in patients with ulceration at baseline. A microbial predictive score (MPS) for MH was developed as a point system, using these six genera. The AUC for predicting MH was 0.80 (sensitivity, 0.64; specificity, 0.92). Multivariate regression analysis demonstrated that MPS was the strongest predictor of MH (OR 37.5 [3.41–411.99]). Patients with a higher abundance of these bacteria were also significantly less likely to relapse over 11–13 months follow-up, with no relapses at the final follow-up in patients scoring ≥5 (0 vs. 40% relapse rate for MPS ≥5 and < 5, respectively). Combining MPS < 5 with serum albumin (threshold <41.5 g/L) differentiated patients further into moderate and high risk of relapse at final follow up (20% vs 40%, respectively). These findings were not related to a specific therapy, but demonstrated that lack of eubiosis may serve as a treatment target for early escalation of therapy.^31^

## Discussion

In this systematic review, we summarized the current literature on microbiome predictors of response to advanced therapies for IBD. The included studies encompass articles reporting baseline or interval microbiome analyses that putatively predicted, or were associated, with a future treatment response. We have highlighted key themes in the literature that may serve as future biomarkers of treatment response, namely, the favorable abundance of fecal SCFA-producing bacteria and metabolic pathways related to butyrate synthesis; the abundance of fecal opportunistic bacteria associated with non-remission; non-butyrate metabolomic predictors from various sample types (including BAs, amino acids, and lipids); and mycobiome predictors of response. Several predictive indices (using baseline or delta values) have been described, demonstrating that microbial predictors of the response to therapy are more accurate than clinical or biochemical biomarkers alone. Only one study to date provides a predictive model, with independent validation, that may enable the selection of one biologic therapy over another.^33^ All other studies present associations that have not been externally validated, and in one study^42^ the predictive score was not specific to a particular therapy. It may be that certain microbial milieus at baseline, or the failure to achieve a specific shift in microbial parameters from baseline, could help us to select patients with more refractory disease who may benefit from enhanced monitoring or early treatment escalation. Further data are required to establish whether this practice would lead to better outcomes. The identification of microbial biomarkers that are most predictive of response would enable targeted analysis, and thus the utilization of less expensive techniques, making it more applicable to everyday clinical care.

Data regarding the mechanisms underlying the proposed associations are largely derived from in vitro and animal studies. SCFAs (acetate, propionate, and butyrate) are produced by several bacteria, particularly Bacillota, of the order Clostridia. SCFAs are a carbon source for colonic epithelial cells and play an important role in mucosal barrier function.^65,66^ Their anti-inflammatory properties include boosting extrathymic production of regulatory T cells in mice,^67^ reduction in oxidative stress via inhibition of NF-kB,^68^ inhibition of IFNɣ,^69^ induction of IL-10 (*F. prausnitzii*),^70^ upregulation of antimicrobial peptides (*Roseburia*)^71^ and they have also been associated with immune surveillance.^66^ *Bifidobacterium*, a SCFA-producing organism associated with response to therapy, has also been linked to mucosal barrier function via promotion and maturation of the colonic epithelium.^72^ However, supplementation of observed microbiome deficiencies has generally not been helpful. Butyrate therapy has not been shown to be efficacious in IBD^73–75^ and there is insufficient evidence that restoration of a dysbiotic microbiome with pro-, pre-, or synbiotic therapy is routinely beneficial.^76^ *Bifidobacterium longum* isolated from the feces of healthy individuals attenuated DSS-induced colitis in mice and augmented the efficacy of IFX. This was associated with an increase in fecal secondary BAs.^77^ However, replenishing bacterial strains known to be relatively deficient in patients with quiescent UC in vivo has not been associated with a reduced risk of flare.^78^

The interaction between BAs, microbiota, and luminal inflammation has been previously reviewed.^79^ Lee and colleagues have corroborated this data and, in addition, demonstrated a link between microbial diversity, BA synthesis, and a favorable response to anti-cytokine over vedolizumab therapy and validated their hypothesis in independent cohorts. This study is the first to demonstrate an association between microbiota composition, metabolomic markers and a differential response to advanced therapy.^33^ Less is understood about the putative mechanistic associations between response to therapy and other metabolites discussed in this manuscript.^28^ Similarly, there is minimal data regarding the mycobiome. A recent RCT randomized consecutive patients with fecal *Candida* (28% of 242 patients screened) with active mild-to-moderate UC to standard therapy plus placebo (*n* = 30) or fluconazole (*n* = 31). The primary endpoint of endoscopic response at 4-weeks was not reached, but secondary endpoints were nominally significant, favoring antifungal therapy (reduction in Robart’s histological score [74% vs. 33%] and fCal [84% vs. 37%]). The presence of fecal *Candida* was associated with baseline disease severity but the groups were poorly matched, with more patients in the placebo group taking corticosteroids at enrollment.^80^ Further studies are required to establish whether fungal dysbiosis is relevant to therapeutic response or simply a marker of disease severity.

### Limitations

The available data are heterogeneous and have several limitations. Many of the included studies were small (some with opposing results^26,29^) and their findings were not validated in independent cohorts. Additionally, the approach to microbiome analysis varies. The majority evaluated the relative, rather than absolute, microbial abundance. The former is influenced by the total microbial count and therefore may be less sensitive in identifying alterations in specific microbes that may be associated with the outcome assessed. Amplicons, rather than whole genome sequencing, assess the hypervariable region of the 16S rRNA gene, which may be unable to identify closely related species or strains sharing similar genetic makeup that may be relevant to disease pathogenesis or trajectory.^23^ The majority of studies incorporate combined cohorts of both UC and CD.^5,14,25,27,29,32–36,38,42,44,45,48^ Large cohort studies with over 500 patients have shown microbiota differences between UC and CD; in particular, reduced diversity in CD with an overall loss of beneficial species such as *Faecalibacterium, Oscillospira, Bifidobacterium* and *Ruminococcus*. ^39^ Differential abundances between UC and CD have also been demonstrated in smaller studies in both the bacteriome and the mycobiome,^32,38,45^ with disease locations^39,81^ and/or phenotype^5^ likely influencing microbial signatures. The degree to which each of these factors may influence microbial predictive markers is unclear, but stratification of the PRISM dataset by IBD type did not alter the predictive value of VedoNet.^42^ Other factors such as cohort age (pediatric vs adult)^82^ as well as sample type (fecal vs mucosal vs saliva)^83^ are also likely to contribute to heterogeneity in results between studies. Once additional studies focusing on pediatric cohorts and sample types other than feces have been undertaken, microbial biomarkers of response which differ between pediatric and adult cohorts as well as fecal and other sample types may be able to be assessed. However, due to the limited pediatric and non-fecal sample studies at this time these comparisons have not been made in this review.

Definitions of response and remission vary significantly with many studies using clinical parameters alone to define response or remission^14,15,30,42,43,49^ which are known to correlate poorly with endoscopic healing.^84^ Where endoscopic assessments have been used at baseline and follow-up, studies are small,^26,29,35^ although one study did achieve baseline and follow-up assessments in 49 CD patients with objective scoring assessed and proposed a predictive score for endoscopic healing at week 30.^40^ In other studies, using endoscopy, there is a lack of clarity regarding the number of patients in whom it was performed^25,28,36,37^ or objective scores were not employed or defined.^35,48^ Two studies did present endoscopic outcomes in reasonably sized cohorts^33,38^ but neither were endoscopically assessed at baseline, without which it is not possible to truly understand the influence of disease severity at baseline, which may confound the results. Other studies used a combination of clinical, biochemical, and/or endoscopic parameters with three, including CSFR, in their definition of response.^27,37,47^ In an area with so much scientific uncertainty, it seems sensible to define remission with the gold standard of endoscopy performed at baseline and follow-up, although this more invasive method may hinder clinical trial enrollment.

Zhou et al performed a meta-analysis combining their Chinese cohort with data from the RISK and PRISM registries (USA) and showed similarities in IBD microbiome profiling across ethnicities.^5^ However, geographical differences in microbiota profiles have previously been described.^85,86^ Potential confounders were not routinely addressed in the included studies (e.g., dietary intake, exercise,^87^ smoking,^88^ alcohol consumption, age, BMI^39^). This is of particular relevance because in the 502 IBD patients investigated by Yilmaz et al, BMI and age exerted more significant changes in microbial composition than any disease or treatment-related factor.^39^ The exclusion of antibiotics prior to enrollment in the majority of studies is perhaps necessary at this stage of our understanding of the microbiome. However, future studies including these groups will be required, since they are commonly used in everyday clinical practice, particularly in CD.

We did not include studies investigating the response to 5-ASA or corticosteroid therapy for the reasons outlined in our methods. Thus, one large multicenter study (PROTECT) investigating predictors of response in newly diagnosed children with UC was excluded.^89^ It is worth mentioning that *Ruminococcaceae* and *Sutterella* were associated with CSFR at week 52 even after adjusting for clinical predictors. In combination with a rectal gene signature (lower antimicrobial peptide gene expression [PC1]), reduced *Suterella* and increased *Ruminococcaceae* at baseline were associated with CSFR at 52 weeks. Based on these data, our results should not be evaluated in isolation. Further work is required to differentiate microbial predictors associated with refractoriness to any therapy in patients who may require more aggressive treatment algorithms, from those in whom particular therapies should be avoided due to the low likelihood of response.

We have also not delved deeply into the mechanisms behind the associations described, which is outside the scope of this review. Several of the included studies also correlated the demonstrated microbial predictors with differential proteomic and genomic findings that will be relevant to future research.^29,30,33,35^ Aside from multiomics, the interaction between microbiome predictors and drug pharmacokinetics deserves further evaluation. Microbial composition has been associated with both IFX^90^ and vedolizumab^27^ levels and in the epi-IIRN database, the use of antibiotics (particularly cephalosporins and penicillins) was associated with immunogenicity to IFX. The latter group replicated increased rates of immunogenicity in antibiotic-exposed mice, while germ-free mice (harboring no microorganisms) did not develop anti-drug antibodies.^91^

### Concluding remarks

Research in this area is challenging, with the key question remaining unanswered: Does the inflammatory environment and associated epithelial disruption lead to dysbiosis, or is microbial dysbiosis the instigator of a pro-inflammatory state? HCs have been frequently enrolled in studies to identify the parameters of health and disease. However, using data derived from HCs to create predictive models^14^ is potentially flawed if patients with IBD are unable to reach eubiosis even in the presence of MH.^48^

Despite the data limitations, we present an updated summary of microbiome predictors of response to advanced therapy in IBD. Future research should focus on collaborative, multiomic analyses with clearly defined homogenous cohorts and definitions of treatment response. New multicentre studies recruiting cohorts in different geographical locations are already underway.^92,93^ The aim would be to move toward interventional studies where predictive scores alter treatment pathways in the hope that we can improve long-term patient outcomes.

## Supplementary Material

Supplemental Material

## Data Availability

All available data is presented in the submitted work.
