# Supplementary material for: Gut microbiome-associated predictors as biomarkers of response to advanced therapies in inflammatory bowel disease: a systematic review
Source: Gut Microbes. 2023 Dec 3;15(2):2287073. doi: 10.1080/19490976.2023.2287073 (PMC10730146; doi:10.1080/19490976.2023.2287073)
Supplement: Supplemental Material [file KGMI_A_2287073_SM4427.docx]

**Supplementary information**

**Supplementary information 1. Search string.**

Ovid MEDLINE(R) ALL <1946 to February 03, 2023>

1 crohn's disease.mp. or exp Crohn Disease/ 62141

2 ulcerative colitis.mp. or exp Colitis, Ulcerative/ 57273

3 inflammatory bowel disease.mp. or exp Inflammatory Bowel Diseases/ 115616

4 1 or 2 or 3 129178

5 exp Antibodies, Monoclonal/ or exp Tumor Necrosis Factor-alpha/ or exp Tumor Necrosis Factor Inhibitors/ or anti-TNF.mp. 402057

6 ustekinumab.mp. or exp Ustekinumab/ 2985

7 exp Antibodies, Monoclonal, Humanized/ or vedolizumab.mp. 83748

8 anti-integrin.mp. 881

9 anti-integrin*.mp. 952

10 exp Adalimumab/ or adalimumab.mp. 10618

11 infliximab.mp. or exp Infliximab/ 17299

12 risankizumab.mp. 349

13 exp Janus Kinase Inhibitors/ or tofacitinib.mp. 3579

14 upadacitinib.mp. 473

15 ozanimod.mp. or exp Sphingosine-1-Phosphate Receptors/ 1133

16 exp Mercaptopurine/ or exp Azathioprine/ or thiopurine.mp. 21688

17 filgotinib.mp. 233

18 methotrexate.mp. or exp Methotrexate/ 59728

19 exp Certolizumab Pegol/ or certolizumab.mp. 1560

20 etrolizumab.mp. 94

21 golimumab.mp. 1575

22 5 or 6 or 7 or 8 or 9 or 10 or 11 or 12 or 13 or 14 or 15 or 16 or 17 or 18 or 19 or 20 or 21 482133

23 microbiome.mp. or exp Microbiota/ 94693

24 exp Genomics/ or genomics.mp. 192797

25 proteomics.mp. or exp Proteomics/ 98620

26 metabolomic.mp. or exp Metabolomics/ 36785

27 multiomics.mp. 1868

28 multi-omic*.mp. 7290

29 gene expression profiling.mp. or exp Gene Expression Profiling/ 165165

30 exp Metagenome/ or exp DNA, Bacterial/ or exp Genomics/ or exp Sequence Analysis, DNA/ or exp Gastrointestinal Microbiome/ or exp Metagenomics/ or metagenomic*.mp. or exp Microbiota/ 551897

31 dysbiosis.mp. or exp Dysbiosis/ 16956

32 metaproteomic.mp. 382

33 metataxanomic.mp. or exp RNA, Ribosomal, 16S/ 64166

34 23 or 24 or 25 or 26 or 27 or 28 or 29 or 30 or 31 or 32 or 33 837104

35 treatment outcome.mp. or exp Treatment Outcome/ 1243979

36 treatment response.mp. 38403

37 therapeutic response.mp. 15260

38 drug efficacy.mp. 7601

39 efficacy.mp. 1015888

40 effectiveness.mp. 574555

41 predict*.mp. 2080352

42 exp Remission Induction/ or remission.mp. 175084

43 35 or 36 or 37 or 38 or 39 or 40 or 41 or 42 4444468

44 4 and 22 and 34 and 43 167

45 enteral nutrition.mp. or exp Enteral Nutrition/ 25826

46 crohn's disease exclusion diet.mp. 34

47 exp Diet/tu, th [Therapeutic Use, Therapy] 104

48 exp Diet Therapy/ or Diet/ 238263

49 diet.mp. or exp Diet, Healthy/ or exp "Diet, Food, and Nutrition"/ or Diet, Macrobiotic/ or exp Diet Therapy/ or exp Diet, Carbohydrate Loading/ or exp Diet/ or exp Diet, Mediterranean/ 2100442

50 crohn's disease exclusion diet.mp. 34

51 CDED.mp. 36

52 exp Dietary Fiber/ or fibre.mp. 63325

53 probiotics.mp. or exp Probiotics/ 34560

54 prebiotic.mp. or exp Prebiotics/ 10724

55 synbiotic.mp. or exp Synbiotics/ 1750

56 fermented food.mp. or exp Fermented Foods/ 33998

57 exp Nutrition Therapy/ or nutrition.mp. 330539

58 exp Dietary Fiber/ or dietary fibre.mp. 24767

59 dietary carbohydrate.mp. or exp Dietary Carbohydrates/ 100078

60 dietary fat.mp. or exp Dietary Fats/ 100376

61 mediterranean diet.mp. or exp Diet, Mediterranean/ 8112

62 elemental diet.mp. or exp Food, Formulated/ 12023

63 specific carbohydrate diet.mp. 55

64 45 or 46 or 47 or 48 or 49 or 50 or 51 or 52 or 53 or 54 or 55 or 56 or 57 or 58 or 59 or 60 or 61 or 62 or 63 2322708

65 4 and 34 and 43 and 64 464

66 44 or 65 607

Embase <1974 to 2023 February 06>

1 inflammatory bowel disease.mp. or exp ulcerative colitis/ or exp inflammatory bowel disease/ or exp Crohn disease/ 211218

2 crohn's disease.mp. or exp Crohn disease/ 117549

3 Ulcerative colitis.mp. or exp ulcerative colitis/ 98707

4 1 or 2 or 3 219176

5 monoclonal antibody.mp. or exp monoclonal antibody/ 767415

6 exp tumor necrosis factor antibody/ or exp adalimumab/ or exp infliximab/ or exp tumor necrosis factor alpha antibody/ or anti-tnf.mp. or exp monoclonal antibody/ 742436

7 golimumab.mp. or exp golimumab/ 9610

8 exp certolizumab pegol/ or certolizumab.mp. 9416

9 exp vedolizumab/ or anti-integrin*.mp. 7969

10 etrolizumab.mp. or exp etrolizumab/ 410

11 ustekinumab.mp. or exp ustekinumab/ 11773

12 exp risankizumab/ or risankizumab.mp. 1218

13 jak inhibitior.mp. 3

14 janus kinase inhibitor.mp. or exp Janus kinase inhibitor/ 27535

15 tofacitinib.mp. or exp tofacitinib/ 8714

16 upadacitinib.mp. or exp upadacitinib/ 1731

17 ozanimod.mp. or exp ozanimod/ 783

18 thiopurine.mp. or exp mercaptopurine/ 29824

19 azathioprine.mp. or exp azathioprine/ 106986

20 exp methotrexate/ or methotrexate.mp. 208020

21 alpha 4 beta 7.mp. 117

22 anti-integrin*.mp. 1432

23 biologic therapy.mp. or exp biological therapy/ 2006868

24 filgotinib.mp. or exp filgotinib/ 997

25 5 or 6 or 7 or 8 or 9 or 10 or 11 or 12 or 13 or 14 or 15 or 16 or 17 or 18 or 19 or 20 or 21 or 22 or 23 or 24 2771798

26 exp microbiome/ or microbiome.mp. or exp bacterial microbiome/ 70071

27 dysbiosis.mp. or exp dysbiosis/ or exp intestine flora/ 100413

28 exp viral metagenomics/ or exp metagenomics/ or metagenomics.mp. 28124

29 exp metabolomics/ or exp multiomics/ or exp proteomics/ or multi-omic*.mp. or exp genomics/ 301639

30 proteomics.mp. or exp functional proteomics/ or exp proteomics/ 141579

31 microbiota.mp. or exp microflora/ 203547

32 exp RNA 16S/ or metataxanomic.mp. 91496

33 exp RNA 16S/ or metataxonomic.mp. or exp intestine flora/ or microflora/ 186738

34 26 or 27 or 28 or 29 or 30 or 31 or 32 or 33 566396

35 treatment response.mp. or exp treatment response/ 346498

36 treatment outcome.mp. or exp treatment outcome/ 2160314

37 exp drug efficacy/ 993296

38 therapeutic response.mp. or exp treatment response/ 333620

39 predict*.mp. 2788254

40 effectiveness.mp. 1066025

41 therapeutic response.mp. or exp treatment response/ 333620

42 predict*.mp. 2788254

43 efficacy.mp. 2073420

44 exp remission/ or remission.mp. 362194

45 35 or 36 or 37 or 38 or 39 or 40 or 41 or 42 or 43 or 44 7174357

46 prebiotic.mp. or exp prebiotic agent/ 16045

47 probiotic.mp. or exp probiotic agent/ 55177

48 fermented food.mp. or exp fermented product/ 46831

49 exp nutrition/ or nutrition.mp. 2628286

50 fibre.mp. or exp fiber/ 128639

51 enteral nutrition.mp. or exp enteric feeding/ 44011

52 exclusive enteral nutrition.mp. 870

53 crohn's disease exclusion diet.mp. 94

54 CDED.mp. 94

55 synbiotic.mp. or exp synbiotic agent/ 3147

56 efficacy.mp. 2073420

57 exp remission/ or remission.mp. 362194

58 exp diet therapy/ or exp low FODMAP diet/ or exp high fiber diet/ or exp liquid diet/ or exp carbohydrate diet/ or exp artificial diet/ or exp low fat diet/ or exp experimental diet/ or exp high calorie diet/ or exp low carbohydrate diet/ or exp low residue diet/ or exp diet/ or exp macrobiotic diet/ or exp protein diet/ or exp full liquid diet/ or exp elemental diet/ or exp gluten free diet/ or exp healthy diet/ or exp Mediterranean diet/ or exp fiber free diet/ or exp low fiber diet/ 713654

59 46 or 47 or 48 or 49 or 50 or 51 or 52 or 53 or 54 or 55 or 58 2854550

60 4 and 25 and 34 and 45 2626

61 4 and 34 and 45 and 59 2025

62 60 or 61 3647

**Supplementary information 2. Data points in pre-defined data capture form**

Data collected included the author, year of publication, study design, primary outcome, number of participants studied (including number with IBD, CD, ulcerative colitis [UC], and microbiome analysis), baseline patient and disease characteristics, assessment time points, definitions of response/remission, objective markers of disease activity, type of microbiome analysis, microbiota (predominantly bacteriome changes [diversity, abundance of SCFA-producing bacteria, opportunistic organisms], but also mycobiome and virome), metabolomic changes, functional analyses, and whether any of the changes described predicted response to therapy.

**Supplementary information 3**. Risk of Bias assessment – Quality assessment tool for before-after (pre-post) studies with no control group

Copied from: https://www.nhlbi.nih.gov/health-topics/study-quality-assessment-tools

| **Criteria**  1. Was the study question or objective clearly stated? | | | |  |  |  |
| --- | --- | --- | --- | --- | --- | --- |
| 2. Were eligibility/selection criteria for the study population prespecified and clearly described? | | | |  |  |  |
| 3. Were the participants in the study representative of those who would be eligible for the test/service/intervention in the general or clinical population of interest? | | | |  |  |  |
| 4. Were all eligible participants that met the prespecified entry criteria enrolled? | | | |  |  |  |
| 5. Was the sample size sufficiently large to provide confidence in the findings? | | | |  |  |  |
| 6. Was the test/service/intervention clearly described and delivered consistently across the study population? |  |  |  |  |  |  |
| 7. Were the outcome measures prespecified, clearly defined, valid, reliable, and assessed consistently across all study participants? | | | |  |  |  |
| 8. Were the people assessing the outcomes blinded to the participants' exposures/interventions? | | | |  |  |  |
| 9. Was the loss to follow-up after baseline 20% or less? Were those lost to follow-up accounted for in the analysis? | | | |  |  |  |
| 10. Did the statistical methods examine changes in outcome measures from before to after the intervention? Were statistical tests done that provided p values for the pre-to-post changes? | | | |  |  |  |
| 11. Were outcome measures of interest taken multiple times before the intervention and multiple times after the intervention (i.e., did they use an interrupted time-series design)? | | | |  |  |  |
| 12. If the intervention was conducted at a group level (e.g., a whole hospital, a community, etc.) did the statistical analysis take into account the use of individual-level data to determine effects at the group level? | | | |  |  |  |

Quality rating by two raters: (Poor, fair, good, cannot determine, not applicable, not reported) – as per recommended NIH guidance.


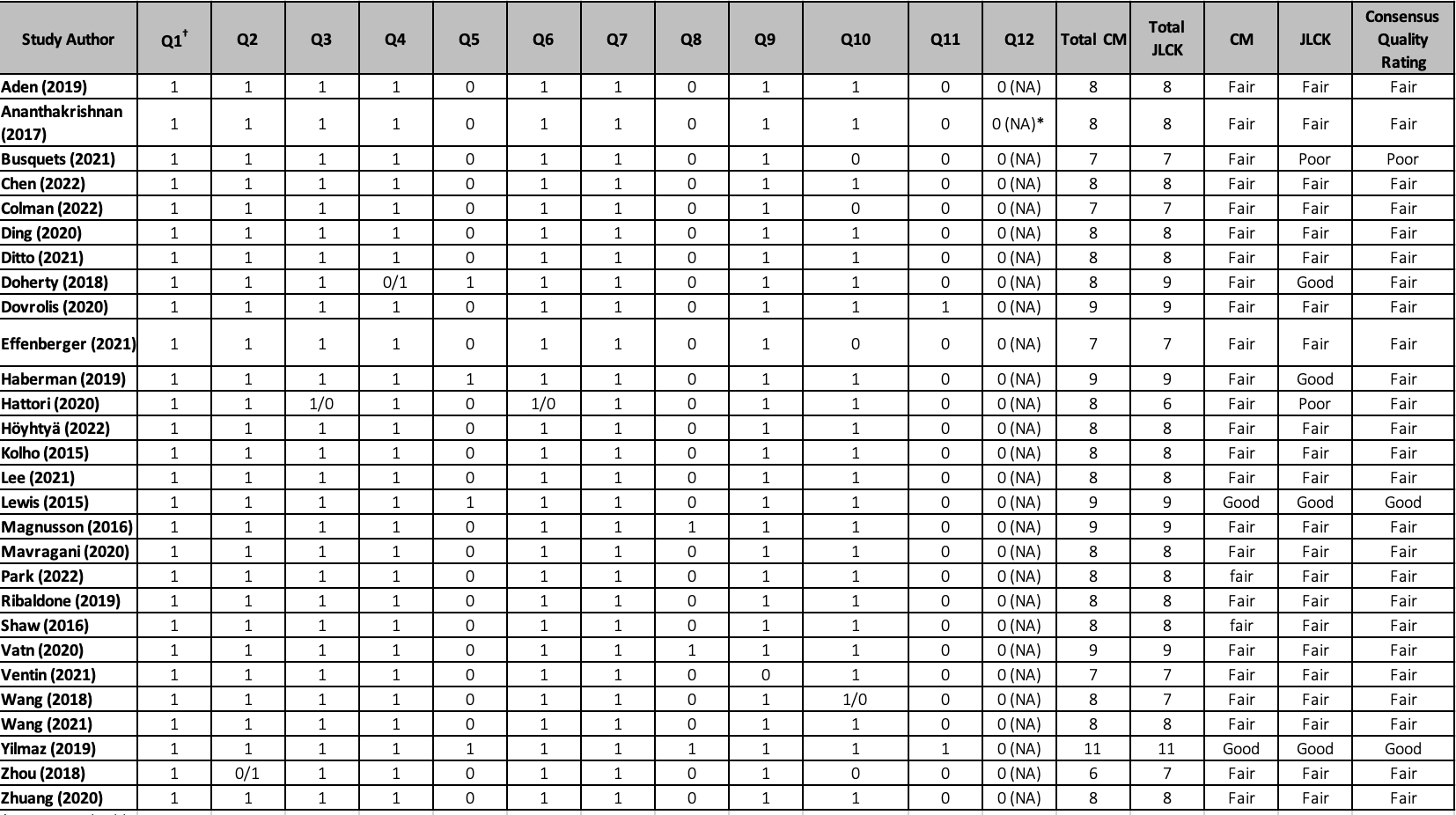


NA,not applicable

**Table S1. Extended summary of included studies**

Includes detailed definitions of response/remission, assessment time points and patient demographic.

| **Author** | **Study type** | **Primary outcome** | **IBD/**  **Total study N** | **Study composition** | **Microbial analysis + responder N** | **Microbial analysis method** | **Assessment time points** | **Therapy** | **Definition of response/remission CD** | **Objective marker of disease activity** | **Responder/Non-responder with microbial analysis (as per study definition)** | **Age** | **Female** | **Disease phenotype** | **Disease duration** | **Bio-naive** | **Exclusions and dietary information** |
| --- | --- | --- | --- | --- | --- | --- | --- | --- | --- | --- | --- | --- | --- | --- | --- | --- | --- |
| **Aden^1^**  2019 | Prospective observational | Microbiome function/structure before and after anti-TNF | 35/170 | Discovery cohort: 12 IBD (8CD, 4 UC), 17 rheumatological, 19 HCs. Validation cohort: 23 IBD (10CD, 13UC) on vedo/anti-TNF & 99 HCs | 35 | Fecal 16s amplicon sequencing, in silico metabolic modelling, inferred metabolomic function from the AGORA resource | Discovery: 0, 2, 6, 30 weeks + single time point from HCs  Validation: 0, 2, 6, 14 weeks + single time point for HCs | anti-TNF – IFX, Etanercept | Clinical response/remission CD: HBI ≤4/decrease in ≥2 points.  Clinical response/remission UC: PMS ≤2, bleeding subscore 0/decrease PMS ≥2 and ≥30%.  Histo: Normal, mild, mod, severe 0-3 | Endoscopy, histology, CRP (NR in whom CRP/endoscopy was performed and neither included in definition of response or remission) | anti-TNF: 15R, 7NR  Vedo: 11R, 2NR  Discovery  9R, 3 NR (Etanercept)  Validation  6R, 4NR (IFX)  11R, 2NR (Vedo) | 38 | 17, 49% | NR | NR | 100% in validation cohort | Dietary intake in subgroup with Potsdam diary |
| **Ananthakrishnan^2^**  2018 | Prospective observational | i) Define relationship between microbial metagenomic structure and function and clinical remission with vedolizumab induction ii) identify microbiome changes on maintenance therapy iii) develop a predictive model of response to therapy | 85/85 | 42 CD, 43 UC | 85 | fecal 16s, Illumina based Shotgun metagenomic sequencing | 0, **14**, 30, 54 weeks | Vedolizumab | Clinical remission at week 14:  CD - HBI <4  UC - SCCAI<2.  Response: Reduction in HBI/SCCAI ≥3 points | CRP but response only defined clinically | 31R 54 NR | 28.5 | 42, 49% | L1 7, L2 15, L3 63; B1 7, B2 15, B3 63; p15; E3 50 | 13 | ‘Majority TNF failures’ | NR |
| **Busquets^3^**  2021 | Prospective observational | Microbial signature of anti-TNF response: R vs PNR <14W) or 2LR (cessation <12m - LOR/AE) | 38/38 | 14 CD, 24 UC  Exclusions:  prior anti-TNF, antibiotics within 1 month, pregnant, severe comorbidity, surgery that compromises intestinal transit | 38 | Fecal analysis of 9 specific species by total DNA qPCR | 0, 1, 2, 3, 6, 9, 12 months | anti-TNF -  8 IFX, 19 ADA, 11 GOLI | CD –  Clinical remission: HBI ≤4  Clinical response: decrease in ≥3 points  Baseline SES-CD (remission not defined)  UC-  Clinical remission: MES ≤1 and/or PMS ≤1  Clinical response: reduction in total Mayo and reduction in rectal bleeding subscore,  ***  Reduction in fCal <250ucg/g  Responders defined as clinical and biological response at 1 year. Non-responders divided into primary non response – no response at week 14 and 2LR (2LR or AEs) | Fcal <250mcg/g.  Unclear as to number of patients who had fcal and endoscopy performed and response was only defined clinically/biochemically. | 31R, 6 NR (combined PNR and 2LR including cessation AEs) | 40.9 | 30, 79% | CD. A1: 0, A2: 9, A3: 5. L1: 6, L2: 2, L3: 6, B1: 12, B2: 1, B3 0, p1. UC E1: 2, E2: 14, E3: 8 | NR | TNF-naive | Prior anti-TNF, antibiotics within 1 month, pregnant, severe comorbidity, surgery that compromises intestinal transit |
| **Chen^4^**  2022 | Retrospectively observational | i) ADA efficacy and safety in Chinese patients for induction and maintenance of remission ii) Characterise fecal microbiota changes during therapy and identify potential predictors of response | 115/115 | 115 CD  Exclusions: pregnancy, lactation, no baseline endoscopy, antibiotics within 4 weeks | 8 | Fecal 16s (Illumina amplicon sequencing) | 0**, 12 weeks** | anti-TNF - ADA | Clinical remission: CDAI <150  Endo response: reduction in CDEIS ≥50%  MH CDEIS 0-3 | Endoscopy – used in definition of response | Not reported in subgroup with microbial analysis | 32.9 | 35, 30.4% | L1 22, L2 18, L3 73, L4 2, B1 91, B2 24, B3 0, p 56 | NR | 27% prior IFX | Pregnancy, lactation, no b/l endoscopy, antibiotics within 4 weeks |
| **Colman^5^**  2022 | Multicentre Prospective and retrospective observational (part of the REFINE cohort) | Primary: To generate a paediatric specific population PK model for vedolizumab to identify patient-specific factors affecting drug clearance as well as evaluating exposure-response relationships. Secondary: explore whether specific microbial signatures are associated with vedolizumab clearance . | 74/74 | 52 CD, 21 UC, 1 IBDU | 13 | Fecal shotgun metagenomic analysis | 0, 2, 14, 26, 52 weeks | Vedolizumab | Prospective: weighted PCDAI, Retrospective; PGA. Combined data stratified into quiescent, mild, moderate or severe disease: wPCDAI ≥12.5, >40, >57.5  CSFR: wPCDAI <12.5 OR quiescent PGA off CS.  Prospective: PUCAI  PUCAI: >10, ≥30, >65.  CSFR: PUCAI<10  quiescent PGA off CS.  FCP remission defined as <250mcg/g | Fcal | 40R week 14, 41R week 52 | 16 | 36, 49% | L1 2, L2 9, L3 41; L4 22; B1 31, B2 9, B3 12; UC/IBDU E4 10/19 | 2.8 | 99% | Enteric infection within 2 weeks of induction |
| **Ding^6^**  2020 | Prospective observational | Metabonomic/metataxonomic predictive markers of response to anti-TNF in CD | 86/99 | 76 CD, 10 UC, 13 HCs | 57 urine, 64 serum, 48 faeces  112 metabolomic patient samples | fecal 16s metagenomic sequencing, metabolites: urine, feces and serum samples via UPLC-MS profiling analyses | 0, 3 monthly samples, predictor for **11-16 months** outcome | anti-TNF – IFX, ADA | CDAI responder – reduction by $\geq$100. Points  Fcal and CRP at baseline in all patients  Response scored out of 7:  CRP decrease by 50% at month or $\leq$5mg/L at month 2-4  Fcal Decrease by 50% at month 2-4 or 4-6 or <50mcg/g at either time point Radiological/endoscopic evidence of improved inflammation | Endoscopy – used in definition of response but number of patients undergoing endoscopy not reported | 11R, 37 NR, 28 partial R | 36.5 | 46, 54% | ND | ND | 100% | Bowel resection |
| **Ditto^7^**  2018 | Prospective observation | Changes in the microbiome at baseline and 6 months after anti-TNF therapy in patients with IBD-associated spondyloarthropathy | 20/20 | 17 CD, 3 UC | 20 | Fecal 16s amplicon sequencing (Illumina)( | 0, **24 weeks** | Anti-TNF -IFX or ADA | CD Response: Decrease in HBI$\geq2$ or $\leq4$ at 6 months with CSFR  UC Response: Decrease in PMS$\geq2$ or $\leq1$ at 6 months with CSFR | Clinical scores only | ND | 53 | 8, 40% | NR | 14.5 | TNF naive | Bowel resection, Pregnancy or  breastfeeding. Inclusion criteria included a Mediterranean diet which all patients followed although it wasn’t specifically evaluated |
| **Doherty^8^**  2018 | RCT (phase 2 CERTIFI data) | Associations between microbiota and response to ustekinumab at 6 weeks | 232/232 | 232 CD | 232 | Fecal 16s amplicon sequencing (Illumina) | 0, 4, 6, 22 weeks | Ustekinumab | Response: $\geq$100 point reduction in CDAI  Remission CDAI<150 | Clinical scores only | 31R, 201 NR | 38 | 146, 63% | ND | 12 | ND | Bowel resection within 6 months, short gut, Clinically significant stricture that could require surgery, Abscess, active TB, infection, cancer |
| **Dovrolis^9^**  2020 | Prospective observational | Microbial profiling and identification of predictors of response to IFX | 20/29 | 14 CD, 6 UC, 9 HCs  Only 10 CD and 4 UC actually received anti-TNF | 14 (28 paired samples, 10 CD, 4 UC) | Mucosal microbiota 16s rRNA amplicon sequencing and gene expression by RT-qPCR. 43 rectal biopsies from 29 patients. 28 pairs pre and post antTNF | 0, 2, 6, 12-20 weeks | anti-TNF - IFX | CD Complete response: HBI <4 normal CRP and complete or near complete MH.  Partial response: Reduction in HBI by >50%but still abnormal, raised CCRP and partial endoscopic healing.  PNR: No change or worsening HBI, CRP or endoscopy  Mayo score  Endoscopy at b/l and 12-20 weeks graded as: complete MH: ulcer resolution; near-complete MH: occasional aphthae/superficial erosions; partial MH: length of inflamed areas shortened but still considerable numbers of persisting ulceration/cobblestone; No MH: lesions worse or similar to baseline  Endoscopy at b/l and 12-20 weeks graded as: complete MH: ulcer resolution; near-complete MH: occasional aphthae/superficial erosions; partial MH: length of inflamed areas shortened but still considerable numbers of persisting ulceration/cobblestone; No MH: lesions worse or similar to baseline | Endoscopy and CRP – used in definition of response | 7R (5CD, 2UC), 7NR (5CD, 2UC) | ND | 3, 21% | A1 1; A2 2 A3 11; L1/2 0; L3 10, B1 9; B2 1 B3 0; E1 0; E2 1; E3 3 | ND | ND | Paeds, antibiotics/probiotics prior 6 weeks, other chronic disease, pregnancy, breastfeeding |
| **Effenberger^10^**  2021 | Prospective observational | To assess longitudinal dynamic changes of gut microbiota in order to  identify a microbial community signature of therapeutic efficacy for azathioprine or anti-TNF | 65/65 | 43 CD, 22 UC  19 CD aza, 24 CD anti-TNF, 10UC aza, 12 UC anti-TNF | 12 aza, 20 anti-TNF | Fecal 16s amplicon sequencing, FastDNA SPIN  Kit  Microbial analysis: 58 | 0, **12, 30 weeks** | Azathioprine, anti-TNF | CD Clinical remission: CDAI<150  +/- deep remission: Fcal <150mcg/g, CRP<5mg/dL.  UC Clinical remission: PMS<2.  +/- deep remission: Fcal <150mcg/g, CRP<5mg/dL.  Deep remission used to define remitters | Fcal, CRP – used in definition | 32R (12 aza, 20 anti-TNF) | 43 | 27, 42% | ND | ND | ND | Antibiotics within 3 months |
| **Haberman^11^**  2019 | Multicentre prospective cohort (PROTECT) | To identify gene expression and microbiota profiles that predict response to therapy in UC | 428/428 | 428 UC  Newly diagnosed, treatment naïve, 206/428 patients underwent rectal biopsy, validation group 50 UC PROTECT and 20 age/sex matched HCs | 152 | Fecal 16s RNA amplicon sequencing + genetic analysis of 206 rectal biopsies, validation in 50 patients. | 0, **4 weeks** | anti-TNF, corticosteroids | Clinical scores  PUCAI <10  Mild: 10-30  Moderate: 35-60 Severe: $\geq$65  Wk 4 remission: PUCAI <10 without treatment escalation or surgery | Baseline endoscopy, then clinically defined scores at follow up | 105R (not reported for subgroup with microbial analysis) | 12.7 | 214, 50% | E2 73, E3 355 | ND | ND | CD, Steroids for alternative condition within 4 weeks, minimum 1-year follow-up. |
| **Hattori^12^**  2020 | Prospective observational | The association between fecal microbiome and SB mucosal healing in SB CD. Secondary endpoint: association between fecal microbiome and relapse | 38/38 | 38 ileal or ileocolonic CD with no active colonic or perianal disease | 38 | Fecal 16s rRNA amplicon sequencing (Illumina) | 0 and **44-54 weeks** | Biologic and immunomodulator (not specified) | MH: absence of ulcers at VCE or DBE where the examination of the entire SB was confirmed and verified by 2 endoscopists. Clinical relapse following endoscopy defined as deterioration of clinical symptoms based on CDAI or positive endoscopic findings requiring treatment change. | Baseline endoscopy then clinically defined scores at follow up. | 14 R, 24 NR | 31.4 | 10, 26% | L1 16, L2 0, L3 22, L4 0, B1 29, B2 9, B3 0 | 10 | ND | Antibiotics within 3 months, colonic inflammation or perianal disease, 64-67% followed elemental diet |
| **Hoyhtya^13^**  2022 | Prospective observational | Aim to determine whether absolute abundance of gut microbes predict response to IFX | 29/29 | 17 CD, 6 UC, 6 IBDU | 29 | Fecal 16s rRNA amplicon sequencing (Illumina): focus on absolute not relative abundance | 0, 2, **6 weeks** | anti-TNF - IFX | Validated symptom score combined with visual analogue scale + fcal <100 = remission (95% NPV), fcal >100 NR | fcal | 10R, 19NR | 14 | 9, 30% | L1 1, L2 4, L3 11, L4Bp 1, UC E3 2, E4 4, IBDU 6 | 1.7 | ND | 1 exclusion (missing fcal); adjusted for antibiotics if received within 1 month |
| **Kolho^14^**  2015 | Prospective observational | Fecal microbiota association with disease activity and therapeutic response to TNF | 68/94 | 36 CD, 26 UC, 6 IBDU, 26 controls (8 HCs, 18 JIA) | 11 (with responder analysis) | DNA extraction from stool and analysed with a phylogenetic microarray (HITChip) | 0, 2, **6, 18 weeks** | anti-TNF – 31 IFX, 1 ADA | PGA, PCDAI, PUCAI, fcal  Responder to anti-TNF: >3fold reduction in fcal or normalisation and mild or no clinical activity | Fcal | 6R, 5NR | 15.5 | 44, 68% | L1 8, L2 5, L3 21, p 2, UC: E2 11, E3 15, IBDU 6 | 15.5 | ND | Diet, antibiotics, probiotics documented; diet included in analysis (similar exclusion between groups) |
| **Lee^15^**  2021 | Prospective observational | Gut metagenome sequencing with serum metabonomics to predict response to biologic therapy | 185/185 | 108 CD, 77 UC | 185 (21 patients included in predictive model analysis) | faecal (metagenomic sequencing, microbial profiling and functional potential) and serum (metabolomics and proteomics) | 0, **14, 54 weeks** | 79 TNF, 21 Uste, 85 Vedo | 14-week clinical remission  52 week clinical and endoscopic remission  CD CR: HBI <3  UC CR: SCCAI<3  ER: Mayo 0-1  ER: SESCD<3 with no ulcers | 89/185 endoscopy at 1 year | Clinical remission  91R at 14 weeks, 113 R at 52 weeks  Endoscopic remission  41R 44NR | ND | ND | ND | ND | ND | Ostomy or pouch |
| **Lewis^16^**  **2015** | Prospective observational | Characterisation of the gut microbiome in paediatric patients initiating therapy with EN or anti-TNF compared to HCs | 90/116 | 90 CD (52 received IFX, 38 EN), 26 HC | 86 (NR EN vs IFX) | Fecal 16s shotgun metagenomic sequencing | 0, 1, 4, 8 weeks | anti-TNF – 50 IFX, 2 ADA, 22 EEN, 16 PEN | Reduction of fcal to below 250mcg/g in those with a level >250ug/g at baseline | Fcal (missing at 8 weeks n=8) | 32R | 13.9 | 28, 54% | L1: 43, L2: 50, L4: 28, p 10. B1: 44, B2: 5, B3: 3, | 0.7 | 100% | Antibiotics in the preceding 6 months |
| **Magnusson^17^**  **2016** | Prospective observational | Determine association between anti-microbial peptides and microbiota profiles in patients with UC before anti-  TNF therapy and correlate these data to treatment outcome | 56 | 56 UC | 7 (at baseline) | Fecal qPCR | 0, 2, **6 weeks** | anti-TNF - 50 IFX, 6 ADA | Clinical response: decrease in total Mayo $\geq$3, | Baseline endoscopy then clinically defined | Faecal microbiota samples in 7 at baseline (4R, 3NR), 15 at wk 2 (8R, 7NR), 13 at week 6 (8R, 5NR); | 36.5 | 16, 28.6% | NR | 3 | 100% | NR |
| **Mavragani^18^**  **2020** | Prospective observational | Explore whether interferon signature affects anti-TNF response via its interactions with microbiome | 30/40 | 22 CD, 8 UC, 10HC | 14 | Amplicon 16s sequencing from colonic mucosal samples qPCR | 0, 1 day prior each drug administration & **12 weeks** | anti-TNF - 24 IFX, 4 ADA, 2 GOLI | HBI, Mayo, CRP, colon wk 0 and 12-20wk.  Remission: HBI<4, normal CRP, complete (no ulcers)/near complete MH (occasional aphthae)  Partial response - drop HBI>50% but >4, abnormal CRP, partial endoscopic response (improved but persistent ulcers), PNR: No change or worsening HBI, CRP, endoscopy | Endoscopy – used in definition of response but without objective score | 7R, 7NR | 40.1 | 11, 37% | L1 5, L2 2, L3 15, B1 19, B2 0, B3 3, E4 8 | ND | ND | Age <18 years, IBDU, malignancy |
| **Park^19^**  **2022** | Prospective observational | Whether microbiome changes at multiple sites can predict the effectiveness of anti-TNF in IBD | 19/39 | 10 CD, 9 UC, 20 HCs | 19 | 16s amplicon sequencing on stool and saliva and also rRNA abundance in extracellular vesicles from feces, saliva, urine, serum | 0, **12 weeks** | anti-TNF - 11 IFX, 5 ADA, 3 GOLI | CD-  Remission: CDAI <150  Mild to moderate: 150-220  Moderate to severe: 220-450 Fulminant: >450  Mayo score  Remission <3  with no subscore >1,  Mild 3-5  Moderate, 6-10 Severe >10  Response: UC $\geq$30% and $\geq$3 point reduction from b/l including rectal bleeding subscore 0-1 or decrease $\geq$1 or $\geq$2 point reduction and 25% in PMS  Response: CDAI reduction in $\geq$70 | Number of patients undergoing endoscopy not clear and objective endoscopic scoring for CD patients not reported | ND | 33 | 6, 32% | ND | ND | TNF naive | Pregnancy, lactation, anti-TNF contraindicated, lack of clinical data or follow-up, antibiotics/probiotics within 3 months |
| **Ribaldone^20^**  **2019** | Prospective observational | Evaluate the microbiota at 6 months of  therapy. (Secondary outcomes: association between microbiome  and CRP at 6 months and predictive  role on response to anti-TNF therapy. | 20/20 | 20 CD | 20 | Faecal 16s rRNA amplicon sequencing | 0 and **>6 months** | anti-TNF - ADA | Response: Decrease in HBI$\geq$2 or HBI $\leq$ 4 at  6 months with ADA drug persistence and no steroid requirement. | CRP but not included in definition of response | 13R, 7NR | 52.5 | 8, 40% | L1 8, L2 12 | 14.5 | 100% | Antibiotics or probiotics in the last month. |
| **Shaw^21^**  **2016** | Prospective observational | Explore longitudinal changes in dysbiosis and ascertain associations between dysbiosis and markers of disease activity and response to therapy | 19/29 | 15 CD, 4 UC, 10 HCs (including 6 unaffected family members) | 17 | Fecal 16s rRNA, amplicon sequencing (Illumina) | 0, **52 weeks**  (5-8 samples per patient) | Immunomodulator or biologic therapy, not defined | PCDAI  PUCAI  FCal,  baseline and follow up colonoscopy at about 12 months. Response determined by MH | Follow up endoscopy defined remission as MH but MH not defined. | 5R, 12NR | ND | ND | ND | 0 | Treatment naive | Prior diagnosis of IBD or prior treatment, history of non-compliance with appointments |
| **Vatn^22^** | Multicentre prospective observational | Identify faecal microbiota signatures associated with IBD and their phenotypes. Secondary outcome: identify signatures associated with disease course and treatment response | 164/324 | 68 CD, 84 UC, 12 IBDU, 116 Non-IBD symptomatic HCs and 44 HCs | 158 (24 with specific outcomes for anti-TNF therapy) | Faecal microbial fluorescent signal strength of 54 predetermined bacterial DNA markers to provide a dysbiosis index | 0, **14 weeks** | Unspecified and anti-TNF | CD -CSFR: HBI <4 &CRP<4  PMS – CSFR: PMS<2 and at least 1 of: fcal<250ucg/g, CRP<4 or MES<2  Severe disease defined by need for treatment escalation to biologic, ciclosporin or surgery. | CSFR with biochemical markers, number of patients with endoscopic scores not reported | Outcome i) Treatment escalation - 117R, 41 NR  ii) anti-TNF response at 14 weeks - 8R, 16NR | 31 | 79, 48% | CD: A1 0, A2 53, A3 15; L1 25, L2 20, L3 23; B1 58, B2 2, B3 7, p4 | 0 | Newly diagnosed, treatment naive | Antibiotics within 1 month, GI infection/other GI auto-immune disease |
| **Ventin-Holmberg^23^**  **2021** | Prospective observational | Predictors of response to IFX in the faecal bacterial and fungal microbiome | 72/72 | 72 | 25 CD, 47 UC | Faecal amplicon sequencing, targeting the  bacterial 16S rRNA gene and fungal ITS 1 region separately | 0, 2, 6, **12,** 52 weeks | anti-TNF - IFX | Endoscopy week 12 (n=59), fcal + clinical score (n=9)  CD:  Remission: SESCD 0-2  Partial remission: SESCD3-6, Non remission SESCD>6  UC/IBDU: total Mayo  Remission: <3 with MES 0-1, partial remission: total mayo 3-4, MES1-2  Non remission: total Mayo $\geq$5 and MES$\geq$2  If no endoscopy  PMS <3 with normal fcal  2 colectomy  If no endoscopy then fcal used. Modified HBI <5 with normal fcal, | Endoscopic end point with biochemical markers used in 9 patients who did not have endoscopy | 44R (13 CD/31UC), 12 partial R (4 CD, 8 UC), 14 NR (6 CD, 8 UC) | 31 | 30, 42% | L1 3, L2 8, L3 14; B1 14, B2 5, B3 6; p 12; E1 1, E2 11, E3 35. | 2 | 100% | If treatment discontinuation for surgery other than colectomy or other reason then microbiome analysis not used |
| **Wang^24^**  **2018** | Prospective observational | Assess faecal microbiota changes during IFX treatment | 11/27 | 11 CD, 16 HCs | 11 (8 baseline samples) | Fecal 16s rRNA amplicon sequencing (Illumina) | 0, after **3-6 doses** | anti-TNF - IFX | Sustained remission: PCDAI $\leq$10 or $\geq$10 point reduction in PCDAI from baseline to after the third infusion or $\leq$10 after the 6th infusion.  Non sustained response PCDAI >10 | Clinical end points only | 4R, 7NR | 11 | 7, 64% | L2 2, L3 8, L4 1 | ND | ND | Antibiotics within last 2 months, other inflammatory condition |
| **Wang^25^**  **2021** | Prospective observational | Explore structure and function of micro/mycobiome and metabolome and their relationship with IFX treatment | 29/49 | 29 CD (18 received IFX), 20 HCs | 18/24 also treated with IFX | Fecal 16s rRNA/fungal ITS amplicon sequencing (Illumina) and targeted metabolomic analysis | 0, after 3-6 doses | anti-TNF - IFX | Sustained remission: PCDAI $\leq$10 or $\geq$10 point reduction in PCDAI from baseline to after the third infusion or $\leq$10 after the 6th infusion.  Non sustained response PCDAI >10 | Clinical end points only | 11R, 7NR | 13 | 12, 66% | L2 2, L3 14, L4 2 | ND | ND | Prebiotics/probiotics or antibiotics within 3 months |
| **Yilmaz^26^**  **2019** | Retrospective observational | Determine microbiota profiles according to disease phenotype, location and severity that are reproducible over the long-term | 502/729 | 270 CD, 232 UC, 229 non IBD | NR (reported as 4500 samples, 5 segments per patient) | Tissue 16s rRNA amplicon sequencing | 5.7 years follow up | anti-TNF | QOL, disease activity, hospitalisation, change in medication, adherence, surgery, CDAI, HBI, MTWAI, SCCAI, fcal, (scores not defined) | Fcal -number of patients with available data not reported | 345R, 157NR | ND | 250, 50% | L1 79 L2 60, L3 97 L4 36, B1 122, B2 75, B3 73, p47,UC E1 30, E2 80, E3 112 | ND | 65% | Insufficient samples |
| **Zhou^27^**  **2018** | Prospective observational (predictors) and cross-sectional study (microbiome associations with IBD vs HCs) | Identify gut microbiome patterns in Chinese IBD patients with different disease activity and status, understand microbiota profiles across different populations, clarify if any microbial biomarkers predict disease progression or response to therapy | 123/196 | 72 CD, 51 UC, 73 HCs (treatment response assessed in 16 CD) | 16 | Fecal 16s rRNA amplicon sequencing (Illumina) | 0, 30 weeks | anti-TNF – IFX | 0 and 30w endoscopy.  CDAI: baseline and at infusions  CRP, ESR, WCC, neutrophil ratio.  Clinical response: $\geq$70 point reduction in CDAI, Clinical remission: CDAI<150  Clinical relapse: CDAI$\geq$150 with an increase $\geq$70 points, the need for steroids, IFX dose escalation or surgery. All other outcomes defined as NR. | Fcal, FBC, CRP, ESR  Endoscopy performed but response clinically defined. | 9R, 7NR | ND | ND | ND | ND | 100% | <18 years old, antibiotics or probiotics within 4 weeks, other chronic disease, pregnancy/breastfeeding |
| **Zhuang^28^**  **2020** | Retrospective observational | Aim to characterise fecal microbiota profiles associated with the clinical and endoscopic response to IFX at week 14 and 30. | 49/49 | 49 CD | 49 | Fecal 16s rRNA amplicon sequencing (Illumina) | 0, 6, **14, 30 weeks** | anti-TNF – IFX | Clinical remission: CDAI <150.  Active disease $\geq$150.  ileocolonoscopy at baseline and 30 weeks. CDEIS by two experienced physicians.  Endoscopic response: $\geq$50% reduction in CDEIS  MH: CDEIS 0-2 | Endoscopy with objective score and used in definition of response. | Clinical remission: 36 (14 weeks), 40 (30 weeks)  MH: 21 (30 weeks)  Endoscopic response: 39 (30 weeks) | 25 | 16, 33% | B1 27, B2 17, B3 5; L1 11, L2 1, L3 37; p 14 | 2 | 100% | <18 years old, antibiotics or probiotics within 4 wks, non-CD IBD, pregnancy/breastfeeding |

*Number of patients with both microbial analysis and responder data. Time points in bold

IBD – inflammatory bowel disease; CD – Crohn’s disease; UC – ulcerative colitis; HBI – Harvey Bradshaw Index; MTWAI – modified Truelove and Witt activity index; SCCAI – simple Crohn’s and colitis activity index; MES – Mayo endoscopic score; PMS – Partial mayo score; ND – Not documented; CDEIS – Crohn’s disease endoscopic index of severity, SES-CD – simple endoscopic score for CD; PGA – physician’s global assessment; MH – mucosal healing; R – response or remission; NR – non-response or non-remission; rRNA – ribosomal ribonucleic acid; anti-TNF – anti-tumour necrosis factor-alpha, IFX – infliximab; ADA -adalimumab, GOLI – golimumab; aza – azathioprine; CDAI – CD disease activity index; (w)PCDAI – (weighted) paediatric CDAI; PUCAI – paediatric UC activity index; fCal – fecal calprotectin; CSFR – corticosteroid free remission; RCT – randomised controlled trial

**Table S2. Reasons for exclusion of studies relevant to the field**

| **Related manuscripts relevant to the literature but excluded** | | |
| --- | --- | --- |
| **Author** | **Year** | **Reason excluded** |
| Busquets^29^ | 2015 | Investigates microbiome changes with therapy, not response to therapy. |
| Lopez-siles^30^ | 2014 | No data on response to therapy in responder/non-responder groups |
| Schierova^31^ | 2021 | No data on response to therapy in responder/non-responder groups |
| Olbjorn^32^ | 2019 | No data on response to therapy in responder/non-responder groups |
| Alatawi^33^ | 2022 | Poorly defined clinical response/overlaps with PNR. Fecal analysis performed at the time of described association (not baseline), therefore not predictive |
| Salamon^34^ | 2020 | Microbial changes during therapy described, not baseline predictors of response |
| Seong^35^ | 2020 | Association with disease activity rather than predicting response to therapy |
| Park^36^ | 2020 | Microbial associations with researcher-assigned baseline prognosis, not response to therapy. |
| Gorelik^37^ | 2021 | Retrospective review in humans associating microbiome with immunogenicity. Prospective data corroborating retrospective data only in mice |
| Gutierrez^38^ | 2016 | Association of bacterial DNA in blood as a risk factor for flare, not response to therapy |
| Rajca^39^ | 2014 | Predictors of relapse not response |
| Sakurai^40^ | 2020 | Predictors of relapse not response |
| He^41^ | 2019 | Association with disease activity before and after treatment irrespective of response to therapy |
| Hyams^42^ | 2019 | Predictors of response but not advanced therapy |
| Kugathasan^43^ | 2017 | Microbial biomarkers for prognostication rather than response/non-response to therapy |
| Sanchis-Artero^44^ | 2021 | Association at time of response, not predictor |
| Kelsen^45^ | 2016 | Oral microbiome at 0 and 8 weeks of therapy in children but no responder data |
| Filip^46^ | 2022 | No responder data |

**Table S3. Microbial and metabolomic analyses performed in each study.**

| Author | Fecal microbiome | Tissue microbiome | Fecal microbial fluorescent signal strength | Extracellular vesicle microbiome analysis | Microbial Metabolites | Functional analysis | Mycobiome | Virome | Predictive model |
| --- | --- | --- | --- | --- | --- | --- | --- | --- | --- |
| Aden^1^ | 1 | 0 | 0 | 0 | 1 | 1 | 0 | 0 | 0 |
| Ananthakrishnan^2^ | 1 | 0 | 0 | 0 | 0 | 1 | 0 | 0 | 1 |
| Busquets^3^ | 1 | 0 | 0 | 0 | 0 | 0 | 0 | 0 | 1 |
| Chen^4^ | 1 | 0 | 0 | 0 | 0 | 1 | 0 | 0 | 0 |
| Colman^5^ | 1 | 0 | 0 | 0 | 0 | 1 | 0 | 0 | 0 |
| Ding^6^ | 1 | 0 | 0 | 0 | 1 | 0 | 0 | 0 | 1 |
| Ditto^7^ | 1 | 0 | 0 | 0 | 0 | 0 | 0 | 0 | 0 |
| Doherty^8^ | 1 | 0 | 0 | 0 | 0 | 0 | 0 | 0 | 0 |
| Dovrolis^9^ | 0 | 1 | 0 | 0 | 0 | 0 | 0 | 0 | 0 |
| Effenberger^10^ | 1 | 0 | 0 | 0 | 0 | 1 | 0 | 0 | 0 |
| Haberman^11^ | 1 | 0 | 0 | 0 | 0 | 0 | 0 | 0 | 1 |
| Hattori^12^ | 1 | 0 | 0 | 0 | 0 | 0 | 0 | 0 | 1 |
| Hoyhtya^13^ | 1 | 0 | 0 | 0 | 0 | 0 | 0 | 0 | 0 |
| Kolho^14^ | 1 | 0 | 0 | 0 | 0 | 0 | 0 | 0 | 1 |
| Lee^15^ | 1 | 0 | 0 | 0 | 1 | 1 | 0 | 0 | 1 |
| Lewis^16^ | 1 | 0 | 0 | 0 | 0 | 0 | 0 | 0 | 0 |
| Magnusson^17^ | 1 | 0 | 0 | 0 | 0 | 0 | 0 | 0 | 0 |
| Mavragani^18^ | 0 | 1 | 0 | 0 | 0 | 0 | 0 | 0 | 0 |
| Park^19^ | 1 | 0 | 0 | 1 | 0 | 0 | 0 | 0 | 0 |
| Ribaldone^20^ | 1 | 0 | 0 | 0 | 0 | 0 | 0 | 0 | 0 |
| Shaw^21^ | 1 | 0 | 0 | 0 | 0 | 0 | 0 | 0 | 1 |
| Vatn^22^ | 0 | 0 | 1 | 0 | 0 | 0 | 0 | 0 | 0 |
| Ventin-Holmberg^23^ | 1 | 0 | 0 | 0 | 0 | 0 | 1 | 0 | 1 |
| Wang^24^ | 1 | 0 | 0 | 0 | 0 | 0 | 0 | 0 | 0 |
| Wang^25^ | 1 | 0 | 0 | 0 | 1 | 0 | 1 | 0 | 0 |
| Yilmaz^26^ | 0 | 1 | 0 | 0 | 0 | 0 | 0 | 0 | 0 |
| Zhou^27^ | 1 | 0 | 0 | 0 | 0 | 0 | 0 | 0 | 1 |
| Zhuang^28^ | 1 | 0 | 0 | 0 | 0 | 1 | 0 | 0 | 1 |

**Table S4. Microbiome and metabolomic predictors of response to therapy**

Summary of outcomes for each study including predictive models.

| Author | Diversity | Predictive of response | Baseline analysis | | | | Predictive Model |
| --- | --- | --- | --- | --- | --- | --- | --- |
|  |  |  | Microbial | Associated with response | Metabolomic and functional analysis | Associated with response |  |
| Aden^1^ | Alpha diversity: Significant trend towards HCs (Chao1, phylogenetic diversity), no change in Shannon index  Beta diversity: Move towards HCs with treatment. Increased interindividual dissimilarity with treatment (Yue and Clayton distance matrix) | No | Coprococcus and Roseburia inulinivorans at baseline in IBD were significantly differed compared to HCs with loss of significance at week 30.  Baseline F. Prausnitzii did not predict response | No | Network modelling: Non remitters vs HCs displayed reduced antagonistic interactions and a baseline reduction of total metabolic interchange even after 30 weeks of treatment.  81% reduction in butyrate synthesis in non-remitters at baseline  Fecal metabolomics N=9 (3CD, 6UC; 5R, 4NR) 0 & 14 wks  At baseline, 3-hydroxyphenylacetic acid was increased in remitters and pyruvic acid in non-remitters. At week 14, butyric acid was significantly increased in remitters and 3-methyl-thioppropionic acid, methyl 2-(methylthio)acetate was increased in non-remitters. | yes | No |
| Ananthakrishnan^2^ | Alpha diversity - Shannon index: Increased at baseline in remitters in CD not UC and only at species level  Beta diversity: Bray Curtis: lower at baseline in CD but not UC remitters. Jaccard: No difference at baseline in remitters vs non-remitters | Possible signal in CD not UC | Differential abundance across taxonomic ranks did not predict response.  *Roseburia inulinivorans* and *Burkholderiales* species higher at baseline in week-14 remitters.  Strain analysis identifying specific SNPs related to L-arginine biosynthesis pathways (in particular amongst *Ruminococcus torques, E. coli* and *Bifidobacterium longus*) were increased in CD patients entering remission at 14 weeks.  Differential abundance in at week 14 ***after*** treatment:  CD remitters: reduced Bifidobacterium longum, Eggerthella, Ruminococcus gnavus and Veillonella parvula.  UC non-remitters: Increased Strepotococcus salivarium. | Yes | At baseline:  CD remitters: increased activity in pathways related to branched chain amino acid, L-citrulline, L-isoleucine, arginine and polyamine synthesis.  UC remitters: reduced activity in pathways related to pentose-phosphate metabolism, pyruvate fermentation to acetate/lactate and N10-formyl-tetrahydrofolate biosynthesis. Increased activity in pathways related colonic acid building blocks biosynthesis and lipid biosynthesis  CD: reduction in activity in 17 metabolic pathways ***after*** treatment at week 14.  15/17 only observed in CD remitters:  Decrease in tricarboxylic acid cyclic (TC) pathways (I and V types) and nicotinamide adenine dinucleotide (NAD) salvage pathway, O-antigen building blocks biosynthesis  CD non-remitters: decrease in L-arginine biosynthesis via N-acetyl-L-citrulline pathway and tetrapyrrole biosynthesis from glutamate pathway  UC remitters: increase in polyamine biosynthesis, non-oxidative pentose phosphate pathway, and sucrose degradation.  UC non remitters: Decrease in gluconeogenesis, uridine monophosphytate (UMP) biosynthesis, and putrescine biosynthesis | yes | ‘ VedoNet’  Modelled in vedolizumab (n=21) and validated in anti-TNF cohort (n=20)  AUC 0.87 (>80% true discovery rate, <25% false discovery rate)  Variables:  Clinical: IBD type, sex, smoking status, age at diagnosis, disease activity at baseline (HBI/SCCAI), disease duration, CRP, ESR, WCC, HB, HCT, PLT, ALB, microbiome composition  Bacterial: *Roseburia_inulinivorans, Bifidobacterium_longum, Ruminococcus_gnavus, Veillonella_parvula, Lactobacillus_salivarius, Eggerthella, Burkholderiales_noname*  and 33 microbiome functional pathways    **Prediction of 14-week remission to vedolizumab.**  Validated in a prospective cohort of 20 patients treated with ant-TNF (14 CD, 6UC), 13 of whom achieved remission at week-14. VedoNET correctly predicted 11/13 cases |
| Busquets^3^ | NR | NA | 9 specific species investigated did no predict response:  *Eubacteria*, *Fecalibacterium Prausnitzii and their 2 phylogroups* (PHGI, PHGII), *Akkermansia muciniphil*, *Escherichia coli, Bacteroidetes*, *Ruminococcus sp. and Methanobrevibacter smithii*.  No individual taxa predicted response but a composite algorithm of relative abundance of 4 microbial markers was predictive of response (Table 4) | Yes | NR | NA | They did not demonstrate response prediction with specific species as predictors of response (9 evaluated) but the ratios of four of these combined together in a model did predict response.  Relative abundance of:  *F. Prausnitzii/Eubacteria;*  *F. Praunitzii phylogroup 1/Eubacteria;*  *Methanobrevibacter smithii/Eubacteria;*  *Ruminococcus/Eubacteria;*  Sensitivity 93.3%, specificity 100%, PPV 100%, NPV 75%  Prediction of anti-TNF response. Unclear at what time point serves as predictor |
| Chen^4^ | Alpha diversity no different at baseline or follow up, no baseline predictor reported (Observed OTU, Shannon Index, Simpson index, Chao I, ACE I)  Beta diversity no difference pre and post treatment.  no baseline predictor reported | No | Changes after treatment but no baseline predictors | No | Microbiome functional analysis revealed upregulation of bgIX (which encodes B-glucosidase involved in starch/sucrose metabolism) and gph (which encodes phosphoglycolate phosphatase involved in glyoxylate and dicarboxylate metabolism) in ADA responders. There was also downregulation of several ATP binding cassette (ABC) transporter genes in the non-responders. | No baseline predictors | No |
| Colman^5^ | Alpha diversity: No prediction at baseline but week 0-2 delta in the Shannon index predicted 14w trough levels which was in turn associated with CSFR.  Bray Curtis: 2 distinct clusters associated with week 14 clinical remission and trough level | Indirect association with variable that is not clearly associated with better treatment outcomes | Increased abundance of *Anaerostipes hadrus* at baseline and *Anaerostipes hadrus, Roseburia Inulinivorans, Faecalibacterium Prausnitzii, Eubacterium rectale* and *Eubacterium eligens* and week 2 predicted a higher week 14 trough vedolizumab level which was associated with week 14 CSFR. | Indirect | Enrichment of butyrate biosynthesis pathways: pyruvate fermentation to butanoate and *Clostridium acetobutylicum* acidogenic fermentation correlated with week 14 vedolizumab trough level | Indirect | No |
| Ding^6^ | NR | NA | Reduced Bacteroidetes with aTNF treatment but no difference in responders/non-responders | No | Analysis in CD only.  Faecal, serum and urinary bile acids differed in responders vs non responders in each fluid with a different specific signature.  Faeces:  increased in responders - Ursocholanic acid conjugated to sulfate and taurine, Diketo-chenodeoxycholic acid isomer conjugated to taurine.  Decreased in responders – Uknown BA conjugated to sulfate and taurine  Serum:  Increased in responders – Diketo-cholic acid conjugated to glycine, deoxycholic acid conjugated to glycine, 3-dehydrocholic acid, unsaturated-chenodeoxycholic acid isomer conjugated to glycine  Decreased in responders – Diketo-chenodeoxycholic acid conjugated to glycine  Urine:  Increase in responders – Ketochenodeoxycholic acid isomer conjugated to N-acetyl-glucosamine  Decrease in responders – Lithocholic acid conjugated to sulfate, Lithocholic acid isomer conjugated to sulfate and glycine, Lithocolic acid isomer conjugated to sulfate and glycine, Chenodeoxycholic acid isomer conjugated to sulfate and glycine  Summary:  CD TNF responders: Increase serum secondary and tertiary bile acids  CD TNF non-responders: increased serum sulphate- and glycine-conjugated primary BAs  Faecal and serum histadine and urinary cysteine also predicted response.  Differential levels of lipid markers:  Decrease in responders -serum phosphatidylcholine, ceramides, sphingomyelins and triglycerides  Increase in responsers: faecal phosphocholines and triglycerides. Faecal lipid profiling provided an AUC of 0.94 (+/-0.10) |  | Predictive metabolomic profiling for CD at 11-16 months after anti-TNF.  Three faecal BAs with strongest association predicted anti-TNF response with AUC of 0.81 (+/-0.17).  Five serum BAs (AUC 0.74 +/- 0.15)  Urine BAs (AUC 0.70 +/- 0.17)  Combination of above did not improve prediction  Urinary cysteine predicting response: AUC 0.78 +/- 0.12.  Fecal/serum histidine poorly predictive  Serum lipids: AUC 0.78 (+/- 0.12); Sn 0.92, Sp 0.61  Faecal lipids: AUC 0.94 (+/- 0.10); Sn 0.81, Sp 0.64 |
| Ditto^7^ | No difference in alpha or beta diversity in responders vs non-responders at baseline | No | Not evaluated according to response | NR | NR | NR | NR |
| Doherty^8^ | Alpha diversity: No difference at baseline in responders at 6 weeks but higher baseline alpha diversity on remitters (Simpson Index).  Differential baseline beta diversity in responders at 6 weeks and baseline community structure different at baseline in 6-week remitters | Yes | No phyla or OTUs at baseline predicted response.  **6-week remitters:** Increased *Faecalibacterium, Bacteroides* and *Ruminococcus*; reduced *E Coli/Shigella* at baseline | Yes | NR | NA | Predicting clinical remission at 6 weeks:  Clinical data: age, sex, baseline steroids, BMI, disease duration and location, fcal, fecal lactoferrin, CRP, bowel stricture, CDAI subscores  Microbial data: OTU relative abundance (*Dialister, Clostridium XI, Coprobacillus, Fuminococcaceae, Ruminococcus, Clostridiales, Coproccoccus, Faecalibacterium, Pasteurellaceae Escherichis/Shigalla*), alpha diversity  AUC  Clinical data: 0.63 (Sp 0.80, Sn 0.45)  Microbial data: AUC 0.84 (Sp 0.77, Sn 0.81)  Combined: AUC (0.84 (Sp 0.83, Sn 0.77)  Predicting clinical response at 6 weeks:  Clinical data: AUC 0.61 (Sp 0.54, Sn 0.72)  Microbial data: AUC 0.76 (Sp 0.56, Sn 0.88)  Combined: AUC 0.73 (Sp 0.72, Sn 0.68)  Post-hoc analysis of 232 patients from CERTIFI, similar in accuracy to using combined predictive models of microbial and clinical data. This was true for both responders and remitters at 6-weeks |
| Dovrolis^9^ | No differences seen in alpha diversity at baseline and in responders/NR.  Difference in Beta diversity at baseline in responders/NR in CD | Beta diversity | **CD responders:** increased at baseline - *Parvimonas* and *Hungatella Roseburia, Ruminococcus_2* and *Stenotrophomonas*  **CD non-responders**: increased at baseline - *Negativibacillus, Faecalibacterium, Eubacterium_hallii_*group*, Blautia, Ruminococcus_gnavus*  **UC non-responders:** increased at baseline – *Sphingomonas*  **UC responders:** increased at baseline - *Sutterella,* *Ruminococcaceae_NK4A214_group, Roseburia, Proteus Oribacterium, Merdibacter,* *Lactobacillus, Lachnospiraceae_NK4A136_group, Lachnospiraceae_ND3007_group, Intestinibacter, Haemophilus, Fournierella, Flavoninfractor, Eubacterium_coprostanoligenes_group* and *Clostridium_sensu_stricto_1*  25 and 23 unique genera predicted response and non-response to IFX, respectively | Yes | NR | NA | No |
| Effenberger^10^ | Shannon index no difference at baseline between remitters/non remitters and no difference after treatment (wk 0>12>30)  PCA Bray curtis no difference at baseline in remitters non remitters. Increased beta diversity longitudinally with treatment in patients with UC who responded to antiTNF but no significance in other groups (CD/aza) | No | Only present longitudinal data, no baseline predictors.  Azathioprine or anti-TNF remitters had a longitudinal reduction in *Proteobacteria* and increase in *Bacteroides*. CD non-remitters had increased abundance of *lactobacillus* over time and *klebsiella* was associated with azathioprine failure. | No | NR | NA | No |
| Haberman^11^ | NR | NA | Taxa associated with disease severity and corticosteroid refractoriness (Increased *Campylobacter, Veillonella, Enterococcus*).  Reduced *Clostridiales* abundance associated with gene signatures that are correlated with severity of disease and treatment non-response | Yes | NR | NA | Model predictive but did not include microbiome variable |
| Hattori^12^ | Reduce Chao I but not Shannon index in the presence of ulcersation but no responder data. | NA | Low relative abundance of *Bacteroidetes* and higher *Fusobacteria* was observed in patients with active endoscopic disease. *Faecalibacterium* (p = 0.008), *Lachnospira* (p = 0.009), *Paraprevotella* (p = 0.01), *Dialister* (p = 0.012, *Streptococcus* (p = 0.025), and *Clostridium* (p = 0.028) were reduced in the ulcer group.  Above genera associated with the presence of ulceration rather than response to therapy per se | NR | NR | NA | A microbial predictive score for mucosal healing was developed as a point system using these six genera. The AUC was 0.80 (sensitivity 0.64, specificity 0.92)  Multivariate regression analysis adjusting for age, sex, smoking history, BMI, CDAI, CRP, ESR albumin, immunomodulator, biologic therapy, elemental diet and PPI demonstrated the microbial composite score to be the strongest predictor for MH (OR 37.5 [3.41-411.99], p=0.003)  Presence of these genera associated with reduced cumulative rate of relapse at 1 year  Patients with a higher abundance of these bacteria were also significantly less likely to relapse over 11-13 months follow up with no relapses at final follow up in patients scoring $\geq$5 (0 vs 40% relapse rate for MPS $\geq$5 and <5 respectively). Combining MPS <5 with serum albumin (threshold <41.5g/L) differentiated patients further into moderate and high risk of relapse at final follow up (20% vs 40% respectively). |
| Hoyhtya^13^ | No difference in alpha or beta diversity at baseline | No | **Remitters at baseline:** Increased absolute and relative abundance of *Bifidobacteriales*, reduced absolute abundance of *Actinomycetales,* increased relative abundance of *Anaerosporabacter*  **Non-remitters at baseline:** Increased in absolute abundance of one species of *Actinomyces* and relative abundance of *Parasutterella* | Yes | NR | NA | No |
| Kolho^14^ | Move towards HCs. No difference at baseline between responders/non-responders but Increased diversity towards HCs was associated normal fcal 3 months later | Improvement in diversity predictive | **Responders:** increased *Bifidobacterium, Clostridium colinum, Eubacterium rectale,* uncultured *Clostridiales* and *Vibrio* and reduced *Streptococcus mitis* at baseline. | Yes | NA | NA | Bidifidobacterium and Clostridium colinum for prediction of response to anti-TNF at 6 weeks: Sn, Sp, PPV, NPV all 1  Eubacterium: Sn, Sp, PPV, NPV all 0.80  Clostridiales Sn 0.83, Sp 1.0, PPV 1.0, NPV 0.8  Strep mitis and Vibrio: 1.0, 0.83, 0.80, 1.0  Not direct comparison of responders with non-responders but the shift towards the eubiosis of HCs at 6 weeks was predictive of biochemical remission (fcal <200ucg/g) 3 months later  Increased Clostiridum sphenoides and Haemophilus spp associated with fcal<200ucg/g 3 months later (AUC 0.88) |
| Lee^15^ | Shannon diversity at baseline did not predict response.  Increased microbial richness at baseline in two metacommunities (rather than microbiome as a whole) predicted clinical (67% vs 36%, p<0.01), but not endoscopic (65% vs 36%, p=0.09) remission at 1 year for anti-cytokine but not anti-integrin therapy. | Yes – microbial richness | Two metacommunities were identified with Group 1 being more likely to respond to anti-cytokine therapy than anti-integrin therapy.  Nine species were associated with response to ustekinumab/anti-TNF therapy: *Clostridium Citroniae, Agathobaculum butyriciproducens, Phascolarctobacterium faecium* [associated with SCFA synthesis], *Dorea, Roseburia, Collinsella stercoris, Bacteroides caccae, Eggerthella lenta, Godonoibacter pamelaeae*.  Three species associated with response to vedolizumab therapy:  *Bifidobacterium longum, Bacteroides stercoris, Bacteroides ovatus* | Yes | Baseline analysis predicting **week 14 response to ustekinumab/anti-TNF** therapy:  **Increased in remitters at baseline:** secondary serum bile acids e.g. glycolithocholate, glycodeoxycholate and ursodeoxycholate  **Increased in non-remitters at baseline:** increased glycerophosphoethanolamines and diacylglycerols  The abundance of secondary bile acids associated with microbiota with 7a/B-deyhdroxylation capacity.  Increased abundance of these microbes in metacommunity 1 associated with response to ustekinumab/anti-TNF therapy.  (validated in 2 independent cohorts). | Yes | 21/185 patients had available clinical, metagenomic, metabolomic and proteomic data  AUC for predicting 14-week response after anti-cytokine therapy 96.3% (95% CI 0.88-1.00). |
| Lewis^16^ | Degree of dysbiosis at baseline (defined as two distinct clusters similar or dissimilar to HCs not correlated with response to therapy | No | NR | NA | NR | NA | No |
| Magnusson^17^ | Lower dysbiosis index at baseline in responders | Yes | **Responders:** Increased levels of F. prausnitzii (both at baseline and up-trending during induction) associated with response.  **Non-responders**: no longitudinal increase in F Prausnitzii was noted during induction. | Yes | NR | NR | NR |
| Mavragani^18^ | NR | NA | NR | NA | Higher baseline serum levels and colonic expression of IFNI and II in 12-week non-responders to anti-TNF. Differential microbial profiles were associated with IFNI and II levels | Indirect association | No |
| Park^19^ | NR | NA | Fecal next generation sequencing (NGS)  **Increased in anti-TNF responders at baseline**: *Actinobacteria, Dorea, Agathobaculum, Blautia*  **Increased in anti-TNF non-responders at baseline:** *Proteobacteria, Enterobacteriaceae, Odoribacter, Ruminococcus gnavus*  Extracellular vesicle stool analysis:  **Increased in responders** *-Firmicutes, Clostridia*, and *Ruminococcaceae.*  **Increased in non-responders:** *Enterobacteriaceae, Acidaminococcaceae*, and *Rikenellaceae*  **Increased in non-remitters:** *Clostridia*  NGS in saliva:  **Increased in responders:** *Abiotrophia defective-spec, FJ976422_s-species*  **Increased in non-responders**: *Ralstonia*  EV saliva:  **Increased in non-responders** – *Prevotella*  *Firmicute/Bacteroidetes* ratio increased in non-responders by 12 weeks but no difference in actual value at baseline or follow-up in responders/non-responders  EV serum:  **Increased in non-responders/non-remitters** – *Corynebacterium*  **Increased in responders at baseline:** *Firmicute/Bacteroidetes* ratio  EV urine: Increased in responders – *Pseudomonadales, Moraxellaceae, Acinetobacter*  Increased in non-responders: *Lachnospiraceae* and *Ruminococcaceae*  *Acidovorax caeni* found in all samples at baseline on EV analysis of responders (and HCs) but not in non-responders | Yes | NR | NA | No |
| Ribaldone^20^ | NR | NA | Differential microbial profiles were demonstrated in CD patients receiving ADA at baseline and 6 months depending on their response to therapy but none predicted response at 6 months | No | NR | NA | No |
| Shaw^21^ | Dysbiosis index at baseline did not predict MH at 1 year. | No | Associations seen on both random forest classifier and generalised estimating equations:  **Reduced in non-responders**: *Coprococcus, Adlercreutzia, Dialister*  **Increased in non-responders:** *Enterobacteriaceae* (unnamed genus) | Yes | NR | NA | Relative abundance of 15 weighted genera, (largely of the Clostridiales family): AUC 76.5%, prediction error in responders (20%) and non-responders (25%).  All 4 UC patients were non-responders |
| Vatn^22^ | NR | NA | No microbial predictors of response to therapy identified | No | NR | NA | No |
| Ventin-Holmberg^23^ | Bacterial diversity higher in responders at 6 weeks but not at baseline. No difference in mycobiome at baseline or throughout treatment.  No difference in richness in terms of response at baseline or throughout treatment for bacteriome or mycobiome | No | Bacteriome  **IBD non-responders:** reduced *Clostridia* (particularly *Ruminococcaceae*)  **IBD non-responders:** increased *Peptostreptococcaceae, Enterobacteriaceae, Granulicatella* at baseline  **IBD responders:** Increased *Odoribacter* and *Ruminococcaceae*  **UC non-responders:** increased *Carnobacteriaceae* and *Bacteroidetes*  **CD responders:** Increased *Bacteroidetes, Bacteroidales, Desulfovibrionales*  **CD non-responders:** Increased *Firmicutes, Bifidobacteriales, Micrococcales, Lactobacillales, Bukholdariales, Pseudomonales*  Mycobiome  **IBD non-responders:** Increased *Candida albicans* at baseline and remained more abundant at 2, 6 and 52 weeks  No difference in *Ascomycota* and *Basidiomycota* ratios at any time point  Correlation between the mycobiome and the bacteriome was also associated with response to therapy | Yes | NR | NA | Selective genera used in a predictive model for 12-week remission:  AUC 0.80 for all IBD patients, 0.84 for CD, 0.79 for UC  Increased accuracy when genera with differential abundance in IBD subtypes included in the model: AUC 0.93 for CD (Bifidobacterium, Rothia, Atopobium, Gemella, Pseudoflavonifractor, Sutterella and Pseudomonas)  AUC for UC 0.82 (Enterococcus, Clostridium, Peptostreptococcus, Faecalibacterium and Candida.) |
| Wang^24^ | NR | NA | Only 4/11 patients treated with IFX maintained remission after up to 6 infusions. Comparison of microbial profiles demonstrated that sustained remission (PCDAI$\leq$10) was associated with a relative increase in SCFA-producing taxa at each time point until final follow-up (*Blautia, Faecalibacterium, Lachnospira* and *Roseburia*) compared with patients who subsequently lost response (PCDAI >10). | Yes | NR | NA | No |
| Wang^25^ | No difference in alpha diversity of micro- or mycobiome | No | Increased in patients with non-sustained response: Clostridium XI, Clostridium XVIII, Eggerthella, Lachnospiracea incertae sedis, Parabacteroides, and Peptococcus.  Increased in patients with sustained response: Methylobacterium, Sphingomonas, Staphylococcus, and Streptococcus (p< 0.05). | Yes | Responders: increased levels of faecal glycine and L-lactic acid.  Non-responders Increased faecal azelaic acid, N-acetylseotonin, sebacic acid, pimelic acid, isovaleric acid, nicotinic acid, adipic acid and 4-aminohippuric acid | Yes | No |
| Yilmaz^26^ | No difference observed | NA | Reduction in CD_A_ cluster associated with worse response to therapy: *Lachnospira, Blautia, Dorea, Coproccous, Ruminococcus, Faecalibacterium, Roseburia, Oscillospira* and *Bilophila*  **CD responders to anti-TNF** (not replicated in steroid group): increased *Bifidobacterium, Collinsella, Lachnospira, Lachnospiraceae, Roseburia, Eggerthella* taxa and reduced *Phascolarctobacterium*  *Eggerthella, Clostridiales* and *Oscillospira* were correlated (in two independent cohorts) with quiescent disease and *Oscillospira* was also associated with a benign disease course. | Yes | NR | NA | No |
| Zhou^27^ | Improved with treatment towards HCs but did not predict response (Shannon index, phylogenetic diversity) | No | CD responders demonstrated normalisation of *Clostridiales* towards that of HCs but no comparison of responders/non-responders. | No | NR | NA | *Clostridiales (Lachnospiraceae), Anaerostipes, Bacteroidales (Bacteriodaceae), Bacteroides,Clostridiales (Veillonellaceae), Veillonella (Dispare), Lactobacillales (Streptococcaceae), Streptococcus (Anginosus)*  Microbial data alone: AUC 87%  Combined with fCal, CDAI: AUC 0.94  CDAI and fCal alone offered accuracy of 59% and 63% |
| Zhuang^28^ | Improved with therapy but did not predict response (Shannon index, OTUs, Chao index).  No change with therapy; Bray Curtis | No | No difference at baseline or week 6 in responders vs non-responders. However, the increase in abundance of *Blautia* and *Lachnospiraceae* from baseline to week 6 predicted clinical response at week 14 and 30. | Yes | KEGG analysis performed with differential pathway enrichment before and after IFX therapy but not assessed for predictive value for response. Pathways involved the following: genetic information processing, biosynthesis of secondary metabolites, cell growth and death, energy metabolism, immune system, nucleotide metabolism, translation, bacterial invasion of epithelial cells and cell cycle-caulobacter | NA | Incremental abundance of *Blautia* and *Lachnospiraceae* from week 6 predicts:  Clinical remission at week 14 (AUC 83% [71-96%]) and week 30 (AUC 84% [72-97%])  Endoscopic efficacy at week 30 (accuracy 89% [79-99%]).  Microbial markers also predicted response better than using clinical data alone.  Combined with albumin and CRP predicts endoscopic response at week 30 (AUC 91% [82-99%]). |

IBD – inflammatory bowel disease; CD – Crohn’s disease; UC – ulcerative colitis; NR – Not reported; NA – not applicable; anti-TNF – anti-tumour necrosis factor-alpha, IFX – infliximab; ADA -adalimumab, GOLI – golimumab CDAI – CD disease activity index; fCal – fecal calprotectin; CDAI – Crohn’s disease activity index; NPV – negative predictive value; PPV – positive predictive value; AUC. – area under the curve; HBI – Harvey Bradshaw Index; SCCAI – Simple Crohn’s and Colitis activity index; CDAI – CD activity index; CRP – C-reactive protein; ESR - Erythrocyte sedimentation rate; WCC – White cell count; Hb – Haemoglobin; HCT – haematocrit; PLT – platelet; ALB – albumin; BA – bile acids; Sn – sensitivity; Sp – specificity

**Table S5: Microbial diversity and prediction of response to therapy**

UC – ulcerative colitis; CD – Crohn’s disease; anti-TNF – anti-tumour necrosis factor-alpha therapy; HC – healthy control, NR – not reported; fCal – fecal calprotectin; PCA – Principal component analysis; OTU – operational taxonomic unit

| **Author** | **Predictive time point** | **Therapy** | **Not reported** | **Did not predict response** | **Predictive of response** |
| --- | --- | --- | --- | --- | --- |
| **Busquets^3^** | 0, 1, 2, 3, 6, 9, 12 months  (Specific time point NR) | anti-TNF -  8 IFX, 19 ADA, 11 GOLI | NR | NA | NA |
| **Ding^6^** | 11 -16 months | anti-TNF – IFX, ADA | NR | NA | NA |
| **Haberman^11^** | 4 weeks | anti-TNF, corticosteroids | NR | NA | NA |
| **Hattori^12^** | 44-54 weeks | Biologic and immunomodulator (not specified) | NR | NA | NA |
| **Mavragani^18^** | 12 weeks | anti-TNF - 24 IFX, 4 ADA, 2 GOLI | NR | NA | NA |
| **Park^19^** | 12 weeks | anti-TNF - 11 IFX, 5 ADA, 3 GOLI | NR | NA | NA |
| **Ribaldone^20^** | >6 months | anti-TNF - ADA | NR | NA | NA |
| **Vatn^22^** | 14 weeks | Unspecified and anti-TNF | NR | NA | NA |
| **Wang 2018^24^** | After 3-6 doses | anti-TNF - IFX | NR | NA | NA |
| **Aden^1^** | Week 14 | anti-TNF – IFX, Etanercept |  | Alpha diversity: Significant trend towards HCs (Chao1, phylogenetic diversity), no change in Shannon index  Beta diversity: Move towards HCs with treatment. Increased interindividual dissimilarity with treatment (Yue and Clayton distance matrix) | No |
| **Chen^4^** | 12 weeks | anti-TNF - ADA |  | Alpha diversity no different at baseline or follow up, no baseline predictor reported (Observed OTU, Shannon Index, Simpson index, Chao I, ACE I)  Beta diversity no difference pre and post treatment.  no baseline predictor reported | No |
| **Ditto^7^** | 24 weeks | Anti-TNF -IFX or ADA |  | No difference in alpha or beta diversity in responders vs non-responders at baseline | No |
| **Effenberger^10^** | Weeks 12 and 30 | Azathioprine, anti-TNF |  | Shannon Index: no difference at baseline between remitters/non remitters and no difference after treatment through to week 30.  PCA Bray Curtis: no difference at baseline in remitters vs non remitters. Increased beta diversity longitudinally with treatment in patients with UC who responded to anti-TNF but no significance in other groups (CD/azathioprine) [no baseline predictor]. | No |
| **Hoyhtya^13^** | 6 weeks | anti-TNF - IFX |  | No difference in alpha or beta diversity at baseline in responders vs non-responders | No |
| **Lewis^16^** | 8 weeks | anti-TNF – 50 IFX, 2 ADA, 22 EEN, 16 PEN |  | Degree of dysbiosis at baseline (defined as two distinct clusters similar or dissimilar to HCs) not correlated with response to anti-TNF therapy | No |
| **Shaw^21^** | 52 weeks | Immunomodulator or biologic therapy, not defined |  | Dysbiosis index at baseline did not predict MH at 1 year. | No |
| **Ventin-Holmberg^23^** | Week 12 | anti-TNF - IFX |  | Bacterial diversity higher in responders at 6 weeks but not at baseline. No difference in mycobiome at baseline or throughout treatment.  No difference in richness in terms of response at baseline or throughout treatment for bacteriome or mycobiome | No |
| **Wang 2021^25^** | After 3-6 doses | anti-TNF - IFX |  | No difference in alpha diversity of micro- or mycobiome | No |
| **Yilmaz^26^** | 5.7 years follow up | anti-TNF |  | No difference observed in alpha diversity in responders/non-responders | No |
| **Zhou^27^** | 30 weeks | anti-TNF – IFX |  | Improved with treatment towards HCs but did not predict response (Shannon index, phylogenetic diversity) | No |
| **Zhuang^28^** | 14 and 30 weeks | anti-TNF – IFX |  | Improved with therapy but did not predict response: Shannon index, OTUs, Chao index  No change with therapy and did not predict response; Bray Curtis | No |
| **Ananthakrishnan^2^** | Week 14 | Vedolizumab |  |  | Possible signal in CD not UC   - Increased alpha diversity (Shannon index) at baseline in CD remitters - Decreased Bray Curtis at baseline in CD remitters |
| **Colman^5^** | 14 weeks | Vedolizumab |  |  | Indirect association with variable that is not clearly associated with better treatment outcomes: Delta alpha diversity 0-2 weeks and Bray Curtis associated with week 14 vedolizumab trough leveI |
| **Doherty^8^** | 6 weeks | Ustekinumab |  |  | Alpha diversity: No difference at baseline in responders at 6 weeks but higher baseline alpha diversity in remitters (Simpson Index).  Differential baseline beta diversity in responders at 6 weeks and differential baseline community structure at baseline in 6-week remitters |
| **Dovrolis^9^** | 12-20 weeks | Anti-TNF - IFX |  |  | No differences seen in alpha diversity at baseline and in responders/NR.  Difference in Beta diversity at baseline in responders/NR in CD |
| **Kolho^14^** | During maintenance, 3 months ahead of analysis | anti-TNF – 31 IFX, 1 ADA |  |  | Improvement in diversity towards HCs predictive.   - No difference at baseline between responders/non-responders but Increased diversity towards HCs at week 6 was associated with fCal <200mcg/g 3-months later |
| **Lee^15^** | 14 and 54 weeks | 79 TNF, 21 Uste, 85 Vedo |  |  | Shannon diversity at baseline did not predict response.  Increased microbial richness at baseline in two metacommunities (rather than microbiome as a whole) predicted clinical (67% vs 36%, p<0.01), but not endoscopic (65% vs 36%, p=0.09) remission at 1 year for anti-cytokine but not anti-integrin therapy. |
| **Magnusson^17^** | 6 weeks | anti-TNF - 50 IFX, 6 ADA |  |  | Lower dysbiosis index at baseline in responders |

**Table S6. Study summary - Bacterial composition, functional analyses and metabolomic predictors of response to therapy**

SNP – single nucleotide polymorphism; CSFR – corticosteroid-free clinical remission; IFN – interferon; EEN – exclusive enteral nutrition; PEN – partial enteral nutrition; anti-TNF – anti-tumour necrosis factor-alpha, IFX – infliximab; ADA -adalimumab, GOLI – golimumab; CD – Crohn’s disease; UC – ulcerative colitis; IBD – inflammatory bowel disease

| **Author** | **Predictive time point** | **Therapy** | **Microbial** | **Metabolomic and functional analysis** |
| --- | --- | --- | --- | --- |
| Microbial and metabolomic associations | | | | |
| **Ananthakrishnan^2^** | Week 14 | Vedolizumab | Differential abundance across taxonomic ranks did not predict response.  *Roseburia inulinivorans* and *Burkholderiales* species higher at baseline in IBD week-14 remitters.  Strain analysis identifying specific SNPs related to L-arginine biosynthesis pathways (in particular amongst *Ruminococcus torques, E. coli* and *Bifidobacterium longus*) were increased in CD patients entering remission at 14 weeks. | At baseline:  **CD remitters:** increased activity in pathways related to branched chain amino acid, L-citrulline, L-isoleucine, arginine and polyamine synthesis.  **UC remitters:** reduced activity in pathways related to pentose-phosphate metabolism, pyruvate fermentation to acetate/lactate and N10-formyl-tetrahydrofolate biosynthesis. Increased activity in pathways related colonic acid building blocks biosynthesis and lipid biosynthesis |
| **Lee^15^** | 14 and 54 weeks | 79 TNF, 21 Uste, 85 Vedo | Two metacommunities were identified with Group 1 being more likely to respond to anti-cytokine therapy than anti-integrin therapy.  Nine species were associated with **response to ustekinumab/anti-TNF therapy**: *Clostridium Citroneae, Agathobaculum butyriciproducens, Phascolarctobacterium faecium* [associated with SCFA synthesis], *Dorea, Roseburia, Collinsella stercoris, Bacteroides caccae, Eggerthella lenta, Godonoibacter pamelaeae*.  Three species associated with **response to vedolizumab therapy:**  *Bifidobacterium longum, Bacteroides stercoris, Bacteroides ovatus* | Baseline analysis predicting **week 14 response to ustekinumab/anti-TNF** therapy:  **Increased in IBD remitters at baseline:** secondary serum bile acids e.g. glycolithocholate, glycodeoxycholate and ursodeoxycholate  **Increased in IBD non-remitters at baseline:** increased glycerophosphoethanolamines and diacylglycerols  The abundance of secondary bile acids associated with microbiota with 7a/B-deyhdroxylation capacity.  Increased abundance of these microbes in metacommunity 1 associated with response to ustekinumab/anti-TNF therapy.  (validated in 2 independent cohorts). |
| **Wang 2021^25^** | After 3-6 doses | anti-TNF - IFX | **Increased at baseline in CD patients with non-sustained response:** *Clostridium XI, Clostridium XVIII, Eggerthella, Lachnospiracea incertae sedis, Parabacteroides, and Peptococcus.*  **Increased at baseline in CD patients with sustained response:** *Methylobacterium, Sphingomonas, Staphylococcus,* and *Streptococcus (p< 0.05).* | **CD responders:** Increased levels of faecal glycine and L-lactic acid.  **CD non-responders:** Increased faecal azelaic acid, N-acetylseotonin, sebacic acid, pimelic acid, isovaleric acid, nicotinic acid, adipic acid and 4-aminohippuric acid |
| Microbial associations | | | | |
| **Busquets^3^** | 0, 1, 2, 3, 6, 9, 12 months  (Specific time point NR) | anti-TNF -  8 IFX, 19 ADA, 11 GOLI | 9 specific species investigated did not predict response:  *Eubacteria*, *Fecalibacterium Prausnitzii and their 2 phylogroups* (PHGI, PHGII), *Akkermansia muciniphil*, *Escherichia coli, Bacteroidetes*, *Ruminococcus sp. and Methanobrevibacter smithii*.  No individual taxa predicted response but a composite algorithm of relative abundance of 4 microbial markers was predictive of response (Table 4) | NR |
| **Hoyhtya^13^** | 6 weeks | anti-TNF - IFX | **Remitters at baseline:** Increased absolute and relative abundance of *Bifidobacteriales*, reduced absolute abundance of *Actinomycetales,* increased relative abundance of *Anaerosporabacter*  **Non-remitters at baseline:** Increased in absolute abundance of one species of *Actinomyces* and relative abundance of *Parasutterella* | NR |
| **Doherty^8^** | 6 weeks | Ustekinumab | No phyla or OTUs at baseline predicted response.  **6-week remitters:** Increased *Faecalibacterium, Bacteroides* and *Ruminococcus*; reduced *E Coli/Shigella* at baseline | NR |
| **Haberman^11^** | 4 weeks | anti-TNF, corticosteroids | Taxa associated with disease severity and corticosteroid refractoriness (Increased *Campylobacter, Veillonella, Enterococcus*).  Reduced *Clostridiales* abundance associated with gene signatures that are correlated with severity of disease and treatment non-response | NR |
| **Kolho^14^** | During maintenance, 3 months ahead of analysis | anti-TNF – 31 IFX, 1 ADA | **Responders:** increased *Bifidobacterium, Clostridium colinum, Eubacterium rectale,* uncultured *Clostridiales* and *Vibrio* and reduced *Streptococcus mitis* at baseline. | NA |
| **Magnusson^17^** | 6 weeks | anti-TNF - 50 IFX, 6 ADA | **Responders:** Increased levels of F. prausnitzii (both at baseline and up-trending during induction) associated with response.  **Non-responders**: no longitudinal increase in F Prausnitzii was noted during induction. | NR |
| **Park^19^** | 12 weeks | anti-TNF - 11 IFX, 5 ADA, 3 GOLI | Fecal amplicon sequencing:  **Increased in anti-TNF responders at baseline**: *Actinobacteria, Dorea, Agathobaculum, Blautia*  **Increased in anti-TNF non-responders at baseline:** *Proteobacteria, Enterobacteriaceae, Odoribacter, Ruminococcus gnavus*  Extracellular vesicle (EV) stool analysis:  **Increased in responders** *-Firmicutes, Clostridia*, and *Ruminococcaceae.*  **Increased in non-responders:** *Enterobacteriaceae, Acidaminococcaceae*, and *Rikenellaceae*  **Increased in non-remitters:** *Clostridia*  Salivary amplicon sequencing:  **Increased in responders:** *Abiotrophia defective-spec, FJ976422_s-species*  **Increased in non-responders**: *Ralstonia*  EV saliva:  **Increased in non-responders** – *Prevotella*  *Firmicute/Bacteroidetes* ratio increased in non-responders by 12 weeks but no difference in actual value at baseline or follow-up in responders/non-responders  EV serum:  **Increased in non-responders/non-remitters** – *Corynebacterium*  **Increased in responders at baseline:** *Firmicute/Bacteroidetes* ratio  EV urine:  **Increased in responders** – *Pseudomonadales, Moraxellaceae, Acinetobacter*  **Increased in non-responders:** *Lachnospiraceae* and *Ruminococcaceae*  *Acidovorax caeni* found in all samples at baseline on EV analysis of responders (and HCs) but not in non-responders | NR |
| **Shaw^21^** | 52 weeks | Immunomodulator or biologic therapy, not defined | Associations seen on both random forest classifier and generalised estimating equations:  **Reduced in non-responders**: *Coprococcus, Adlercreutzia, Dialister*  **Increased in non-responders:** *Enterobacteriaceae* (unnamed genus) | NR |
| **Dovrolis^9^** | 12-20 weeks | Anti-TNF - IFX | **CD responders:** increased at baseline - *Parvimonas* and *Hungatella Roseburia, Ruminococcus_2* and *Stenotrophomonas*  **CD non-responders**: increased at baseline - *Negativibacillus, Faecalibacterium, Eubacterium_hallii_*group*, Blautia, Ruminococcus_gnavus*  **UC non-responders:** increased at baseline – *Sphingomonas*  **UC responders:** increased at baseline - *Sutterella,* *Ruminococcaceae_NK4A214_group, Roseburia, Proteus Oribacterium, Merdibacter,* *Lactobacillus, Lachnospiraceae_NK4A136_group, Lachnospiraceae_ND3007_group, Intestinibacter, Haemophilus, Fournierella, Flavoninfractor, Eubacterium_coprostanoligenes_group* and *Clostridium_sensu_stricto_1*  25 and 23 unique genera predicted response and non-response to IFX, respectively | NR |
| **Ventin-Holmberg^23^** | Week 12 | anti-TNF - IFX | Bacteriome  **IBD non-responders:** reduced *Clostridia* (particularly *Ruminococcaceae*)  **IBD non-responders:** increased *Peptostreptococcaceae, Enterobacteriaceae, Granulicatella* at baseline  **IBD responders:** Increased *Odoribacter* and *Ruminococcaceae*  **UC non-responders:** increased *Carnobacteriaceae* and *Bacteroidetes*  **CD responders:** Increased *Bacteroidetes, Bacteroidales, Desulfovibrionales*  **CD non-responders:** Increased *Firmicutes, Bifidobacteriales, Micrococcales, Lactobacillales, Bukholdariales, Pseudomonales*  Mycobiome  **IBD non-responders:** Increased *Candida albicans* at baseline and remained more abundant at 2, 6 and 52 weeks  No difference in *Ascomycota* and *Basidiomycota* ratios at any time point  Correlation between the mycobiome and the bacteriome was also associated with response to therapy | NR |
| **Wang 2018^24^** | After 3-6 doses | anti-TNF - IFX | Only 4/11 patients treated with IFX maintained remission after up to 6 infusions. Comparison of microbial profiles demonstrated that sustained remission (PCDAI$\leq$10) was associated with a relative increase in SCFA-producing taxa at each time point until final follow-up (*Blautia, Faecalibacterium, Lachnospira* and *Roseburia*) compared with patients who subsequently lost response (PCDAI >10). | NR |
| **Yilmaz^26^** | 5.7 years follow up | anti-TNF | Reduction in CD_A_ cluster associated with worse response to therapy: *Lachnospira, Blautia, Dorea, Coproccous, Ruminococcus, Faecalibacterium, Roseburia, Oscillospira* and *Bilophila*  **CD responders to anti-TNF** (not replicated in steroid group): increased *Bifidobacterium, Collinsella, Lachnospira, Lachnospiraceae, Roseburia, Eggerthella* taxa and reduced *Phascolarctobacterium* | NR |
| **Zhuang^28^** | 14 and 30 weeks | anti-TNF – IFX | No difference at baseline or week 6 in responders vs non-responders. However, the increase in abundance of *Blautia* and *Lachnospiraceae* from baseline to week 6 predicted clinical response at week 14 and 30. | KEGG analysis performed with differential pathway enrichment before and after IFX therapy but not assessed for predictive value for response. Pathways involved the following: genetic information processing, biosynthesis of secondary metabolites, cell growth and death, energy metabolism, immune system, nucleotide metabolism, translation, bacterial invasion of epithelial cells and cell cycle-caulobacter |
| **Colman^5^** | 14 weeks | Vedolizumab | Increased abundance of *Anaerostipes hadrus* at baseline and *Anaerostipes hadrus, Roseburia Inulinivorans, Faecalibacterium Prausnitzii, Eubacterium rectale* and *Eubacterium eligens* at week 2 predicted a higher week 14 trough vedolizumab level which was associated with week 14 CSFR. | Functional analysis demonstrated enrichment of butyrate biosynthesis pathways: pyruvate fermentation to butanoate and *Clostridium acetobutylicum* acidogenic fermentation. These correlated with week 14 vedolizumab trough level |
| Metabolomic associations | | | | |
| **Aden^1^** | Week 14 | anti-TNF – IFX, Etanercept | *Coprococcus* and *Roseburia inulinivorans* at baseline in IBD were significantly differed compared to HCs with loss of significance at week 30.  Baseline *F. Prausnitzii* did not predict response | Network modelling:  Non remitters vs HCs displayed reduced antagonistic interactions and a baseline reduction of total metabolic interchange even after 30 weeks of treatment.  **IBD non-remitters at baseline:** 81% reduction in butyrate synthesis  Fecal metabolomics N=9 (3CD, 6UC; 5RM, 4NRM) 0 & 14 weeks  **Increased in remitters at baseline:** 3-hydroxyphenylacetic acid  **Increased in non-remitters at baseline:** pyruvic acid |
| **Ding^6^** | 11 -16 months | anti-TNF – IFX, ADA | Reduced *Bacteroidetes* with anti-TNF treatment but no difference in responders/non-responders and no predictors of response identified | Analysis in CD only.  Faecal, serum and urinary bile acids differed in responders vs non responders in each fluid with a different specific signature.  Faeces:  **increased in responders** - Ursocholanic acid conjugated to sulfate and taurine, Diketo-chenodeoxycholic acid isomer conjugated to taurine.  **Decreased in responders** – Unknown bile acid conjugated to sulfate and taurine  Serum:  **Increased in responders** – Diketo-cholic acid conjugated to glycine, deoxycholic acid conjugated to glycine, 3-dehydrocholic acid, unsaturated-chenodeoxycholic acid isomer conjugated to glycine  **Decreased in responders** – Diketo-chenodeoxycholic acid conjugated to glycine  Urine:  **Increase in responders** – Ketochenodeoxycholic acid isomer conjugated to N-acetyl-glucosamine  **Decrease in responders** – Lithocholic acid conjugated to sulfate, Lithocholic acid isomer  conjugated to sulfate and glycine, Lithocolic acid isomer conjugated to sulfate and glycine, Chenodeoxycholic acid isomer conjugated to sulfate and glycine  Summary (bile acids):  **CD anti-TNF responders:** Increase serum secondary and tertiary bile acids  **CD anti-TNF non-responders:** increased serum sulphate- and glycine-conjugated primary bile acids  Faecal and serum histidine and urinary cysteine increased in responders.  Differential levels of lipid markers:  **Decrease in responders** -serum phosphatidylcholine, ceramides, sphingomyelins and triglycerides  **Increase in responders:** faecal phosphocholines and triglycerides. |
| **Mavragani^18^** | 12 weeks | anti-TNF - 24 IFX, 4 ADA, 2 GOLI | NR | Higher baseline serum levels and colonic expression of IFNI and II in 12-week non-responders to anti-TNF. Differential microbial profiles were associated with IFNI and II levels |
| No baseline predictors of response | | | | |
| **Chen^4^** | 12 weeks | anti-TNF - ADA | Changes after treatment (increased in CD responders: *Barnesiella, Anaerostipes, Tyzzerella, Lachnoclostridium, and Lachnospiraceae_unclassified*) but no baseline predictors | Microbiome functional analysis revealed upregulation of bgIX (which encodes B-glucosidase involved in starch/sucrose metabolism) and gph (which encodes phosphoglycolate phosphatase involved in glyoxylate and dicarboxylate metabolism) in ADA responders. There was also downregulation of several ATP binding cassette (ABC) transporter genes in the non-responders. |
| **Effenberger^10^** | Weeks 12 and 30 | Azathioprine, anti-TNF | Only present longitudinal data, no baseline predictors.  Azathioprine or anti-TNF remitters had a longitudinal reduction in *Proteobacteria* and increase in *Bacteroides*. CD non-remitters had increased abundance of *lactobacillus* over time and *klebsiella* was associated with azathioprine failure. | NR |
| **Ribaldone^20^** | >6 months | anti-TNF - ADA | Differential microbial profiles were demonstrated in CD patients receiving ADA at baseline and 6 months depending on their response to therapy but none predicted response at 6 months | NR |
| **Vatn^22^** | 14 weeks | Unspecified and anti-TNF | No microbial predictors of response to therapy identified | NR |
| **Zhou^27^** | 30 weeks | anti-TNF – IFX | CD responders demonstrated normalisation of *Clostridiales* towards that of HCs but no comparison of responders/non-responders. | NR |
| **Ditto^7^** | 24 weeks | Anti-TNF -IFX or ADA | Not evaluated according to response | NR |
| **Hattori^12^** | 44-54 weeks | Biologic and immunomodulator (not specified) | Low relative abundance of *Bacteroidetes* and higher *Fusobacteria* was observed in patients with active endoscopic disease. *Faecalibacterium* (p = 0.008), *Lachnospira* (p = 0.009), *Paraprevotella* (p = 0.01), *Dialister* (p = 0.012, *Streptococcus* (p = 0.025), and *Clostridium* (p = 0.028) were reduced in the ulcer group.  Above genera associated with the presence of ulceration rather than response to therapy per se | NR |
| **Lewis^16^** | 8 weeks | anti-TNF – 50 IFX, 2 ADA, 22 EEN, 16 PEN | NR | NR |

**References**

1. Aden K, Rehman A, Waschina S, et al. Metabolic Functions of Gut Microbes Associate With Efficacy of Tumor Necrosis Factor Antagonists in Patients With Inflammatory Bowel Diseases. Gastroenterology 2019;157:1279-92.e11.

2. Ananthakrishnan AN, Luo C, Yajnik V, et al. Gut Microbiome Function Predicts Response to Anti-integrin Biologic Therapy in Inflammatory Bowel Diseases. Cell Host Microbe 2017;21:603-10.e3.

3. Busquets D, Oliver L, Amoedo J, et al. RAID Prediction: Pilot Study of Fecal Microbial Signature With Capacity to Predict Response to Anti-TNF Treatment. Inflamm Bowel Dis 2021;27:S63-s6.

4. Chen L, Lu Z, Kang D, et al. Distinct alterations of fecal microbiota refer to the efficacy of adalimumab in Crohn's disease. Front Pharmacol 2022;13:913720.

5. Colman RJ, Mizuno T, Fukushima K, et al. Real world population pharmacokinetic study in children and young adults with inflammatory bowel disease discovers novel blood and stool microbial predictors of vedolizumab clearance. Aliment Pharmacol Ther 2022.

6. Ding NS, McDonald JAK, Perdones-Montero A, et al. Metabonomics and the Gut Microbiome Associated With Primary Response to Anti-TNF Therapy in Crohn's Disease. J Crohns Colitis 2020;14:1090-102.

7. Maria Chiara Ditto, Simone Parisi, Gianpiero Landolfi, et al. Intestinal microbiota changes induced by TNF-inhibitors in IBD-related spondyloarthritis. RMD Open 2021;7:e001755.

8. Doherty MK, Ding T, Koumpouras C, et al. Fecal Microbiota Signatures Are Associated with Response to Ustekinumab Therapy among Crohn's Disease Patients. mBio 2018;9.

9. Dovrolis N, Michalopoulos G, Theodoropoulos GE, et al. The Interplay between Mucosal Microbiota Composition and Host Gene-Expression is Linked with Infliximab Response in Inflammatory Bowel Diseases. Microorganisms2020.

10. Effenberger M, Reider S, Waschina S, et al. Microbial Butyrate Synthesis Indicates Therapeutic Efficacy of Azathioprine in IBD Patients. Journal of Crohn's and Colitis 2021;15:88-98.

11. Haberman Y, Karns R, Dexheimer PJ, et al. Ulcerative colitis mucosal transcriptomes reveal mitochondriopathy and personalized mechanisms underlying disease severity and treatment response. Nature Communications 2019;10:38.

12. Hattori S, Nakamura M, Yamamura T, et al. The microbiome can predict mucosal healing in small intestine in patients with Crohn's disease. J Gastroenterol 2020;55:1138-49.

13. Höyhtyä M, Korpela K, Saqib S, et al. Quantitative Fecal Microbiota Profiles Relate to Therapy Response During Induction With Tumor Necrosis Factor α Antagonist Infliximab in Pediatric Inflammatory Bowel Disease. Inflammatory Bowel Diseases 2022:izac182.

14. Kolho KL, Korpela K, Jaakkola T, et al. Fecal Microbiota in Pediatric Inflammatory Bowel Disease and Its Relation to Inflammation. Am J Gastroenterol 2015;110:921-30.

15. Lee JWJ, Plichta D, Hogstrom L, et al. Multi-omics reveal microbial determinants impacting responses to biologic therapies in inflammatory bowel disease. Cell Host Microbe 2021;29:1294-304.e4.

16. Lewis JD, Chen EZ, Baldassano RN, et al. Inflammation, Antibiotics, and Diet as Environmental Stressors of the Gut Microbiome in Pediatric Crohn's Disease. Cell host & microbe 2015;18:489-500.

17. Magnusson MK, Strid H, Sapnara M, et al. Anti-TNF Therapy Response in Patients with Ulcerative Colitis Is Associated with Colonic Antimicrobial Peptide Expression and Microbiota Composition. J Crohns Colitis 2016;10:943-52.

18. Mavragani CP, Nezos A, Dovrolis N, et al. Type I and II Interferon Signatures Can Predict the Response to Anti-TNF Agents in Inflammatory Bowel Disease Patients: Involvement of the Microbiota. Inflamm Bowel Dis 2020;26:1543-53.

19. Park YE, Moon HS, Yong D, et al. Microbial changes in stool, saliva, serum, and urine before and after anti-TNF-α therapy in patients with inflammatory bowel diseases. Sci Rep 2022;12:6359.

20. Ribaldone DG, Caviglia GP, Abdulle A, et al. Adalimumab Therapy Improves Intestinal Dysbiosis in Crohn's Disease. J Clin Med 2019;8.

21. Shaw KA, Bertha M, Hofmekler T, et al. Dysbiosis, inflammation, and response to treatment: a longitudinal study of pediatric subjects with newly diagnosed inflammatory bowel disease. Genome Med 2016;8:75.

22. Vatn S, Carstens A, Kristoffersen AB, et al. Faecal microbiota signatures of IBD and their relation to diagnosis, disease phenotype, inflammation, treatment escalation and anti-TNF response in a European Multicentre Study (IBD-Character). Scand J Gastroenterol 2020;55:1146-56.

23. Ventin-Holmberg R, Eberl A, Saqib S, et al. Bacterial and Fungal Profiles as Markers of Infliximab Drug Response in Inflammatory Bowel Disease. Journal of Crohn's and Colitis 2021;15:1019-31.

24. Wang Y, Gao X, Ghozlane A, et al. Characteristics of Faecal Microbiota in Paediatric Crohn's Disease and Their Dynamic Changes During Infliximab Therapy. J Crohns Colitis 2018;12:337-46.

25. Wang Y, Gao X, Zhang X, et al. Microbial and metabolic features associated with outcome of infliximab therapy in pediatric Crohn's disease. Gut Microbes 2021;13:1-18.

26. Yilmaz B, Juillerat P, Øyås O, et al. Microbial network disturbances in relapsing refractory Crohn’s disease. Nature Medicine 2019;25:323-36.

27. Zhou Y, Xu ZZ, He Y, et al. Gut Microbiota Offers Universal Biomarkers across Ethnicity in Inflammatory Bowel Disease Diagnosis and Infliximab Response Prediction. mSystems 2018;3.

28. Zhuang X, Tian Z, Feng R, et al. Fecal Microbiota Alterations Associated With Clinical and Endoscopic Response to Infliximab Therapy in Crohn's Disease. Inflamm Bowel Dis 2020;26:1636-47.

29. Busquets D, Mas-de-Xaxars T, López-Siles M, et al. Anti-tumour Necrosis Factor Treatment with Adalimumab Induces Changes in the Microbiota of Crohn's Disease. J Crohns Colitis 2015;9:899-906.

30. Lopez-Siles M, Martinez-Medina M, Busquets D, et al. Mucosa-associated Faecalibacterium prausnitzii and Escherichia coli co-abundance can distinguish Irritable Bowel Syndrome and Inflammatory Bowel Disease phenotypes. International Journal of Medical Microbiology 2014;304:464-75.

31. Schierova D, Roubalova R, Kolar M, et al. Fecal Microbiome Changes and Specific Anti-Bacterial Response in Patients with IBD during Anti-TNF Therapy. Cells 2021;10.

32. Olbjørn C, Cvancarova Småstuen M, Thiis-Evensen E, et al. Fecal microbiota profiles in treatment-naïve pediatric inflammatory bowel disease – associations with disease phenotype, treatment, and outcome.

. Clin Exp Gastroenterol 2019;12:37-49.

33. Alatawi H, Mosli M, Saadah OI, et al. Attributes of intestinal microbiota composition and their correlation with clinical primary non-response to anti-TNF-α agents in inflammatory bowel disease patients. Bosnian journal of basic medical sciences 2022;22:412-26.

34. Salamon D, Gosiewski T, Krawczyk A, et al. Quantitative changes in selected bacteria in the stool during the treatment of Crohn's disease. Advances in Medical Sciences 2020;65:348-53.

35. Seong G, Kim N, Joung JG, et al. Changes in the Intestinal Microbiota of Patients with Inflammatory Bowel Disease with Clinical Remission during an 8-Week Infliximab Infusion Cycle. Microorganisms 2020;8.

36. Park SK, Kim HN, Choi CH, et al. Differentially Abundant Bacterial Taxa Associated with Prognostic Variables of Crohn's Disease: Results from the IMPACT Study. J Clin Med 2020;9.

37. Gorelik Y, Freilich S, Gerassy-Vainberg S, et al. Antibiotic use differentially affects the risk of anti-drug antibody formation during anti-TNFα therapy in inflammatory bowel disease patients: a report from the epi-IIRN. Gut 2022;71:287-95.

38. Gutiérrez A, Zapater P, Juanola O, et al. Gut Bacterial DNA Translocation is an Independent Risk Factor of Flare at Short Term in Patients With Crohn's Disease. Am J Gastroenterol 2016;111:529-40.

39. Rajca S, Grondin V, Louis E, et al. Alterations in the intestinal microbiome (dysbiosis) as a predictor of relapse after infliximab withdrawal in Crohn's disease. Inflamm Bowel Dis 2014;20:978-86.

40. Sakurai T, Nishiyama H, Sakai K, et al. Mucosal microbiota and gene expression are associated with long-term remission after discontinuation of adalimumab in ulcerative colitis. Scientific Reports 2020;10:19186.

41. He C, Wang H, Liao W-D, et al. Characteristics of mucosa-associated gut microbiota during treatment in Crohn's disease. World journal of gastroenterology 2019;25:2204-16.

42. Hyams JS, Davis Thomas S, Gotman N, et al. Clinical and biological predictors of response to standardised paediatric colitis therapy (PROTECT): a multicentre inception cohort study. Lancet 2019;393:1708-20.

43. Kugathasan S, Denson LA, Walters TD, et al. Prediction of complicated disease course for children newly diagnosed with Crohn's disease: a multicentre inception cohort study. Lancet 2017;389:1710-8.

44. Sanchis-Artero L, Martínez-Blanch JF, Manresa-Vera S, Cortés-Castell E, Rodriguez-Morales J, Cortés-Rizo X. Evaluation of Changes in Gut Microbiota in Patients with Crohn’s Disease after Anti-Tnfα Treatment: Prospective Multicenter Observational Study. International Journal of Environmental Research and Public Health2020.

45. Kelsen J, Bittinger K, Pauly-Hubbard H, et al. Alterations of the Subgingival Microbiota in Pediatric Crohn's Disease Studied Longitudinally in Discovery and Validation Cohorts. Inflamm Bowel Dis 2015;21:2797-805.

46. Rob F, Schierova D, Stehlikova Z, et al. Association between ustekinumab therapy and changes in specific anti-microbial response, serum biomarkers, and microbiota composition in patients with IBD: A pilot study. PLoS One 2022;17:e0277576.
